# Centromere defects, chromosome instability, and cGAS-STING activation in systemic sclerosis

**DOI:** 10.1038/s41467-022-34775-8

**Published:** 2022-11-18

**Authors:** Souren Paul, Mark H. Kaplan, Dinesh Khanna, Preston M. McCourt, Anjan K. Saha, Pei-Suen Tsou, Mahek Anand, Alexander Radecki, Mohamad Mourad, Amr H. Sawalha, David M. Markovitz, Rafael Contreras-Galindo

**Affiliations:** 1 The Hormel Institute, University of Minnesota, Austin, MN USA; 2 Department of Internal Medicine, University of Michigan, Ann Arbor, MI USA; 3 University of Michigan Scleroderma Program, Division of Rheumatology, Department of Internal Medicine, University of Michigan, Ann Arbor, MI USA; 4 Medical Scientist Training Program, University of Michigan, Ann Arbor, MI USA; 5 Program in Cancer Biology, University of Michigan, Ann Arbor, MI USA; 6 Departments of Pediatrics and Medicine and Immunology and Lupus Center of Excellence, University of Pittsburgh School of Medicine, Pittsburgh, PA USA; 7 Program in Cell and Molecular Biology, University of Michigan, Ann Arbor, MI USA; 8 Program in Immunology, University of Michigan, Ann Arbor, MI USA; 9 Masonic Cancer Center, University of Minnesota, Minneapolis, MN USA

**Keywords:** Cytogenetics, Rheumatic diseases, Centromeres, Skin diseases

## Abstract

Centromere defects in Systemic Sclerosis (SSc) have remained unexplored despite the fact that many centromere proteins were discovered in patients with SSc. Here we report that lesion skin fibroblasts from SSc patients show marked alterations in centromeric DNA. SSc fibroblasts also show DNA damage, abnormal chromosome segregation, aneuploidy (only in diffuse cutaneous (dcSSc)) and micronuclei (in all types of SSc), some of which lose centromere identity while retaining centromere DNA sequences. Strikingly, we find cytoplasmic “leaking” of centromere proteins in limited cutaneous SSc (lcSSc) fibroblasts. Cytoplasmic centromere proteins co-localize with antigen presenting MHC Class II molecules, which correlate precisely with the presence of anti-centromere antibodies. CENPA expression and micronuclei formation correlate highly with activation of the cGAS-STING/IFN-β pathway as well as markers of reactive oxygen species (ROS) and fibrosis, ultimately suggesting a link between centromere alterations, chromosome instability, SSc autoimmunity, and fibrosis.

## Abstract (web summary)

Fibrosis of the skin plays an important role in scleroderma. Here the authors demonstrate genetic and epigenetic abnormalities at the centromere that affect the replication of the chromosomes, resulting in activation of pathways involved in inflammation and fibrosis

## Introduction

Systemic sclerosis (SSc or scleroderma), is a devastating disease that strikes women more commonly than men and generally has its onset between the ages of 30 and 50 years old. SSc leads to induration of the skin, particularly on the arms, legs, neck, face, as well as peripheral calcinosis and Raynaud’s syndrome^1–4^. SSc is subclassified into limited cutaneous (lcSSc) and diffuse cutaneous (dcSSc) based on skin involvement. While both are very disabling illnesses, the latter has higher morbidity and mortality due to significant skin and joint involvement as well as greater degree of visceral involvement, including fibrosis of the esophagus (and other parts of the gastrointestinal track), lungs, and layers of the heart^2–4^. The cause of SSc remains elusive, although genetic predisposition^5^, epigenetic interactions^6,7^, reactive oxidizing species (ROS)^8^, and environmental factors likely play important roles^9^.

The pathophysiology of SSc includes immune activation, vascular injury, and aberrant vasculopathy culminating in fibrosis, a thickening of the connective tissue produced by excessive accumulation of extracellular matrix (ECM) proteins, including collagen type-I and type-III^10^. Activation of fibroblasts, the resident cells of the connective tissue, is a key contributor to fibrosis in SSc^10^. After tissue injury, active fibroblasts migrate to the ECM. In an environment where resident and immune cells secrete a surplus of growth factors, such as fibroblast growth factor (FGF), transforming growth factor β (TGFB1), and interleukins 1 and 6, active fibroblasts differentiate into secretory myofibroblasts in a process called fibroblast to myofibroblast transition^10,11^. Accumulation of a large number of myofibroblasts is responsible for the excessive synthesis of ECM proteins^10–13^.

SSc diagnosis relies on clinical evaluation accompanied by screening for antinuclear antibodies (ANAs)^14,15^. Anti-Topoisomerase-I antibodies (ATAs) (usually called anti-Scl-70) are found predominately in dcSSc patients (prevalence 20–40%), and are associated with poor prognosis, pulmonary fibrosis, and disease progression^14–16^. Anticentromere antibodies (ACAs) are found in up to 43% of lcSSc, previously known as the CREST variant (CREST: calcinosis, Raynaud’s phenomenon, esophageal dysmotility, sclerodactyly, and telangiectasia)^16^. ACAs recognize a large proportion of the centromere proteins (CENPA, CENPB, CENPC, and so on, to CENPT) and, indeed, most of the key proteins involved in centromere formation were discovered using antibodies from SSc patients^16^. ACAs are associated with a generally more favorable prognosis as compared with positivity for ATAs^14–16^, but patients with the former have a high predilection for pulmonary arterial hypertension. Anti-RNA polymerase I and III antibodies are detected in 10–25% of dcSSc patients and are used as a predictive marker of rapid onset of the disease, skin thickening and renal crisis^2–4^. Despite their clinical value for prognosis, the etiology of ATAs and ACAs in SSc patients is unknown.

The centromere is the structural unit responsible for the correct segregation of chromosomes. Destabilization of centromere function results in chromosome instability (CIN), a hallmark of birth defects, cancers, and fibrosis^17–20^. The centromere has two key functions; (i) assemble the kinetochore, and (ii) maintain sister chromatids together before chromosome separation^21,22^. Defects in either of these key functions result in lagging chromosomes, aneuploidy, and micronuclei, all of which are CIN biomarkers^23,24^. CENPA, a H3-histone variant that is only deposited at functional centromeres, is the key epigenetic factor that determines “centromere identity” by defining the location of kinetochore formation in the chromosome^21^. On the other hand, CENPB, which recognizes and binds directly to DNA sequences in centromeres (CENPB boxes), enhances centromere function and its interaction with H3K9me3 maintains cohesion between sister chromatids^22^. Despite the presence of ACAs in SSc, the role that centromere sequences and proteins play in the pathogenesis of SSc remains unexplored.

Centromere DNA sequences are composed of 171 bp α-satellite repeat units organized in a head-to-tail fashion to form arrays of high order repeats (HORs), which can extend for several megabases^25^. During human evolution α-repeats became homogeneous in each centromere core, and today the α-repeat content within a given array is 98–100% similar but is only ~75% similar to other arrays^26,27^. The centromere core is delineated by 1 or 2 large α-repeat-arrays^26^. Towards the periphery (the pericentromere) the sequence is more diverse, with smaller arrays or monomers of alphoid and other repeats, as well as transposon-like elements^28–30^.

Centromeres have been difficult to study, and because of their repetitive sequences genomic assembly has not yet been accomplished, except for the centromeres of chromosomes X and Y^31,32^. We recently developed PCR assays that quantitatively estimate the length of each centromere, save that of the centromere core of chromosome 19, based on the idea that the 171 bp repeats in each centromere are rather unique^33,34^. Assays for pericentromere sequences of chromosome 19 were also developed^33^. These assays enabled us to study the dynamics of centromere length in cancer and congenital disorders, yielding a dynamic assessment of centromere genetics^33,34^. In view of the presence of ACAs in lcSSc, the abnormal cell proliferation of fibroblasts leading to the deposition of collagen, and sporadic reports of chromosomal defects in SSc^35–40^, we investigated whether centromeric DNA alteration and CIN play a role in SSc pathogenesis. Here we show that the DNA-sensing cGAS-STING pathway, known to drive immune activation and inflammation^41,42^, is linked to centromere alterations and CIN in SSc.

## Results

### Centromere genetic defects in SSc fibroblasts

**Fig. 1:**
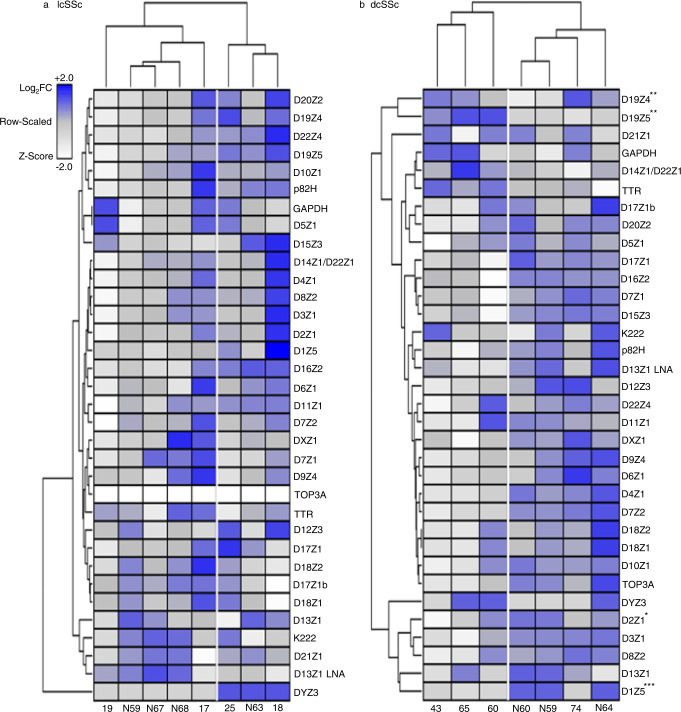
Centromere size variation in primary fibroblasts from patients with SSc. **a** A heatmap depicting alpha-satellite content (rows) obtained by qPCR in 50 ng of DNA from fibroblasts obtained from healthy individuals (N59, N63, N67, and N68) and patients with lcSSc (17, 18, 19, 25). Unsupervised hierarchical clustering analysis was conducted to ascertain if distinct genetic signatures distinguish healthy fibroblasts from lesion fibroblasts. Normal samples are noted to cluster with diseased samples, suggesting that centromeric alterations in fibroblasts obtained from patients with lcSSc do not diverge from variations that occur at baseline in fibroblasts obtained from health individuals. **b** A heatmap depicting alpha-satellite content in fibroblasts from healthy individuals (N59, N60, and N64) and patients with dcSSc (43, 60, 65, and 74). With the exception of the dcSSc sample 74, diseased samples are distinct genetically at the centromeric locus as compared to healthy samples (N59, N60, and N64) and show evidence of marked loss of centromere repeats in multiple chromosomes. The color gradient bar represents relative abundance (left). No significant variation of chromosomal arm gene copy numbers GAPDH, TTR, and TOP3A were observed. The nomenclature of these α-satellites begins with the letter D, followed by their chromosome number (1–22, X or Y), followed by a Z, and a number indicating the order in which these sequences were discovered. The DYZ3 repeat accurately represents the gender of the individuals (light gray: female; blue: male). Data obtained in three replicates were analyzed using a multiple unpaired *t*-test, assuming a gaussian distribution. Stars indicate statistically significant differences between the groups in a *t*-test analysis (*p = < 0.5, **p = < 0.1, ***p = < 0.001). LNA lock nucleic acid. Source Data are provided as a Source Data file.

We investigated the genomic landscape of centromeres in fibroblasts from skin lesions of patients with SSc. The demographics of the patients recruited in this study are shown in Supplementary Table 1. We measured the length of centromeres in healthy forearm skin fibroblasts from age-matched controls and lesion skin fibroblasts from lcSSc and dcSSc patients using our rapid-PCR centromere assay as previously described^33,34^ (Fig. 1). This methodology was previously validated with data obtained by Southern blot, FISH and Next Generation Sequencing (NGS)^33^. As a baseline, we observed significant genomic alterations in several centromere arrays in both healthy subjects and SSc patients (Fig. 1), which correspond to the centromere length differences observed among human populations^33^. Further analysis by type of disease showed deletions and insertions in the centromeres of fibroblasts from lcSSc lesions, but these changes clustered with those seen in healthy fibroblasts, suggesting that these changes were simply part of natural variation (Fig. 1a). In contrast, deletions were found to dominate the centromeric DNA landscape of fibroblasts from patients with dcSSc, which clustered away from healthy fibroblasts (Fig. 1b). In particular, we observed significant deletions in centromere arrays D1Z5 and D2Z1 from chromosomes 1 and 2, respectively, as well as specific centromere expansion in the pericentromeric arrays of Chr 19, D19Z4 and D19Z5 (Fig. 1b). We did not observe significant changes in the chromosomal arm genes GAPDH, TTR or TOP3A. Centromere alterations found in dcSSc patients do not appear to be the result of immunosuppressive treatment, as similar centromere alterations were observed in untreated dcSSc patients (Supplementary Table 1). Taken together, we found that centromeric genetic loss in fibroblasts from skin lesions is prevalent in dcSSc but not in lcSSc, regardless of previous medical treatment.

We also examined centromeres in peripheral blood cells from SSc patients to compared them to the centromere length of skin lesion fibroblasts (Supplementary Figs. 1 and 2). Heatmap analysis of peripheral blood lymphocytes (PBLs) and monocyte-derived-macrophages (MDMs) shows that these blood cells cluster away from fibroblasts in both types of SSc (Supplementary Figs. 1 and 2). Consistent with the above results, both fibroblasts and blood cell populations showed gain or loss of centromeric DNA in lcSSc (Supplementary Fig. 1). In contrast, loss of centromeric DNA was the dominant feature of dcSSc when fibroblasts were compared to peripheral blood cells from the same patient (Supplementary Fig. 2). These results support the idea that the centromere defects observed in dcSSc fibroblasts are not produced from cytotoxic medications, as such centromere abnormalities were not present in peripheral blood cells. Thus, it appears that loss of centromere DNA in lesion skin fibroblasts is a central feature of dcSSc, but not of lcSSc.

Although analysis of centromeric DNA was performed in freshly isolated skin fibroblasts cultured for a brief period of time (~3 passages), we investigated whether centromere DNA changes could be the result of cell passaging. Similarly, we explored whether treatment of the fibroblasts with the antifungal agent Amphotericin B, commonly used during tissue preparation, could affect centromere sequences. We cultured fibroblasts from healthy skin and SSc skin lesions for 3 to 10 passages, some of them treated with Amphotericin B, and quantitated the length of the centromeres using our qPCR centromere assays (Supplementary Fig. 3). The assays showed no significant changes in the length of centromeres or copy number of chromosomal arm genes under these conditions (Supplementary Fig. 3), suggesting that centromere genetic changes observed in SSc fibroblasts were not attributable to passaging in culture or treatment with an antifungal agent.

### Cytogenetic alterations in SSc fibroblasts

**Fig. 2:**
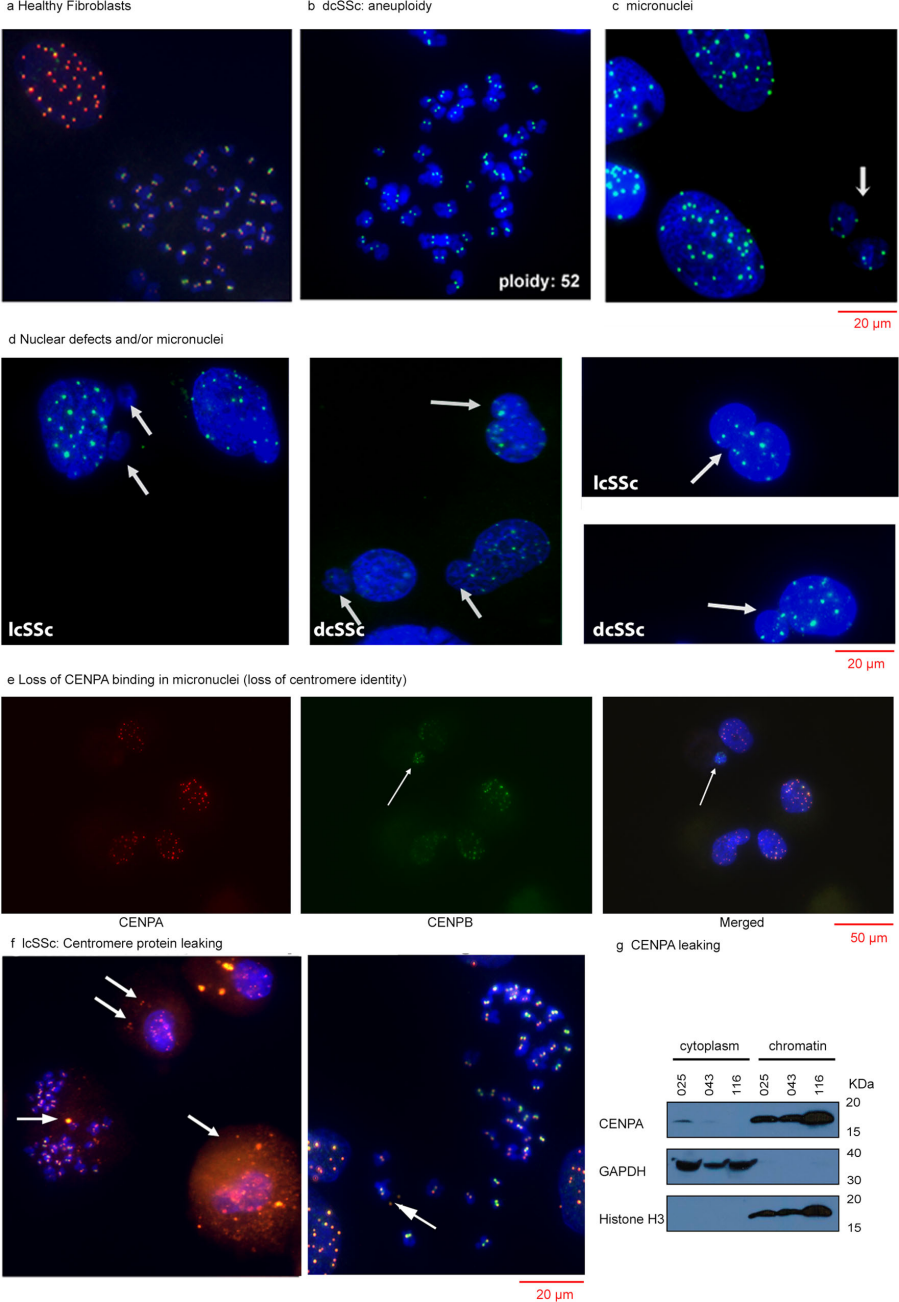
Cytogenetic analysis of SSc skin fibroblasts. Fibroblasts from skin biopsies of healthy individuals (**a**) and SSc patients (**b**–**g**) were arrested in metaphase with colchicine. Chromosomal spreads were stained with anti-CENPA (red) or anti-CENPB (green) antibodies. (In **b**, **c**, and **f** only anti-CENPB). The nuclei/chromosomes were counterstained with DAPI. Shown are representative pictures of at least 10 micrographs. **a** Healthy fibroblasts showing normal ploidy, **b** Aneuploidy in dcSSc fibroblasts showing 52 chromosomes, **c** Micronuclei in SSc fibroblasts (arrows). **d** Nuclear defects and micronuclei (arrows) in dcSSc and lcSSc. **e** Loss of centromere identity in micronuclei from SSc fibroblasts. The arrow indicates a micronucleus stained with CENPB but not CENPA antibodies. **f** Cytoplasmic centromere proteins (arrows) in fibroblasts from ACA-positive lcSSc patients. Yellow indicates colocalization of CENPA and CENPB. **g** Western blotting analysis of CENPA, GAPDH and H3K9Me3 in cytoplasmic and chromatin fractions from SSc fibroblasts grown in the presence of colchicine. GAPDH (cytoplasmic) and H3K9Me3 (chromatin) confirmed the specificity and purity of the fractions. CENPA was detected mostly in nuclear chromatin fractions as expected but was found leaked into the cytoplasmic fraction in lcSSc patient 025, who has ACAs. Patients 043 and 116 with dcSSc are seronegative for ACAs. A faint band is seen in the cytoplasmic fraction in patient 043. A more quantitative description of the data is found in Supplementary Table 1.

Having studied the genetics of centromeres in SSc fibroblasts and identified centromere instability in multiple patients, we evaluated centromere epigenetic marks that modulate centromere function and could lead to CIN when defective. We performed immunofluorescence (IF) on metaphase spreads of SSc fibroblasts using CENPA and CENPB antibodies. Fibroblast spreads from four healthy individuals show normal karyotypes (46 chromosomes) and the expected nuclear/centromere staining of CENPA or CENPB (Fig. 2a, and Supplementary Fig. 4). Strikingly, fibroblasts from 10 of 11 dcSSc patients showed abnormal karyotypes (aneuploidy) in ~10–50% of the chromosome spreads analyzed (Fig. 2b, Supplementary Table 1). We did not see abnormal karyotypes in the fibroblasts of 9 lcSSc patients (Supplementary Table 1). We observed evidence of CIN or chromosome segregation defects in the form of micronuclei in all lcSSc and dcSSc fibroblasts, but not in fibroblasts from healthy individuals (Fig. 2c, d, Supplementary Table 1). Nuclear deformities, as evidenced by morphologies other than the spherical and ovoid forms, were also found in fibroblasts from all dcSSc and lcSSc patients (Fig. 2d, Supplementary Table 1). These findings were almost universal and did not correlate with the presence of antinuclear antibodies, or the therapy received by the patients. The number of micronuclei correlated with the modified Rodnan Skin Score (mRSS) of the patients (*r* = 0.6167, *p* < 0.0143, see below in Supplementary Fig. 13). However, since the fibroblasts were sampled from one site and the mRSS reflects multiple areas, it remains to be determined whether the number of micronuclei found is related to overall disease severity.

### SSc patients show loss of centromere identity in micronuclei

After discovering chromosome segregation defects in SSc fibroblasts, we evaluated centromere marks in fibroblasts isolated from skin lesions of SSc patients. We found that micronuclei showed specific staining with CENPB antibodies, CENPB being a protein that binds to specific centromere DNA sequences called CENPB boxes^22^. However, eukaryotic centromeres are marked epigenetically by nucleosomes containing CENPA, a protein that defines centromere identity and leads to proper kinetochore formation^21,43^. Of the micronuclei that showed CENPB staining, we identified several that were not stained with CENPA (Fig. 2e). Micronuclei with loss of centromere identity were observed in several SSc fibroblast cultures, regardless of subtype (Supplementary Table 1). Thus, we link centromere dysfunction to chromosome segregation defects in lcSSc and dcSSc patients.

### Centromere cytogenetics in blood monocytes and lymphocytes

We next performed cytogenetic evaluations in peripheral blood monocytes cultured in vitro to differentiate into monocyte-derived macrophages (MDM), as well as lymphocytes stimulated with phytohemagglutinin (PHA) from several SSc patients obtained in this study, both for 7 days. We did not find centromere defects, cytogenetic abnormalities, cytoplasmic CENP staining, nor micronuclei (Supplementary Tables 2 and 3). These results indicate that centromere defects found in the fibroblasts from skin lesions of patients with SSc are not found in all cell types and are thus not directly inherited at the genetic level. We hypothesize that patients with SSc might have centromeres that are inherently less stable and so, when exposed to insults such as reactive oxygen species (ROS) (see below) become damaged, although this hypothesis requires future exploration.

### Colocalization of CENPA and CENPB with MHC molecules

In 6 of 9 lcSSc patient fibroblast cultures, we detected centromere/kinetochore mislocalization to the cytoplasm (~4–78%, *n* = 500, Supplementary Table 1), as visualized by cytoplasmic staining of the nuclear protein CENPA (Fig. 2f, and Supplementary Fig. 5). CENPB, a protein that binds directly to CENPB boxes in centromere sequences^22^, was also found in the cytoplasm and colocalized with CENPA (Fig. 2f and Supplementary Fig. 5). Interestingly, cytoplasmic staining of CENPA and CENPB correlated 100% with the detection of ACAs in these patients (Supplementary Table 1, *X*^2^ = 1.00, *p* < 0.0001). These centromere/kinetochore mislocalization defects were not observed in fibroblasts isolated from healthy controls, dcSSc patients, or lcSSc patients lacking ACAs. We also confirmed the finding of cytoplasmic CENPA by examining nuclear and cytoplasmic fractions of lcSSc fibroblasts taken from patients with ACAs. Western blot analysis showed that CENPA is present in cytoplasmic fractions of fibroblasts from lcSSc patients with ACAs, but CENPA was only detected in the nuclear fraction of patients lacking ACAs (Fig. 2g and Supplementary Fig. 6). These results indicate that centromere protein mislocalization is unique to ACA-positive lcSSc patients.

**Fig. 3:**
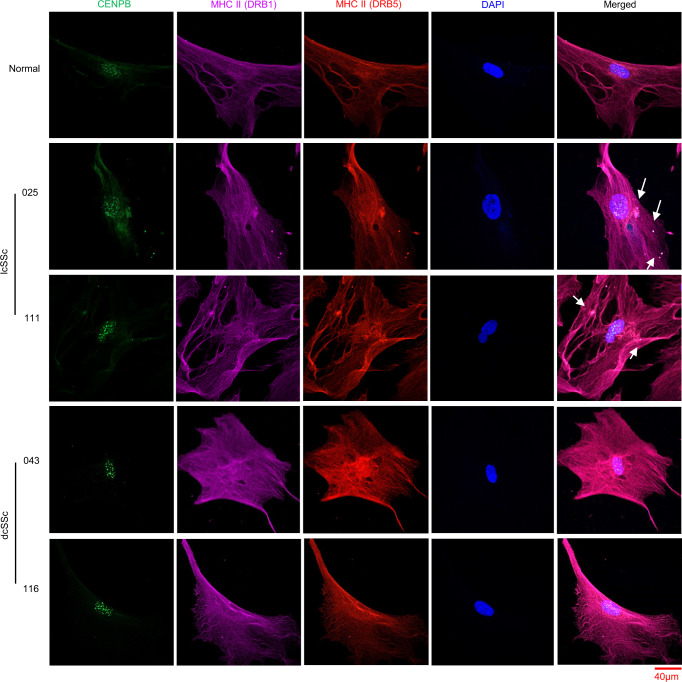
Colocalization of cytoplasmic/membrane CENPB and MHC Class II molecules in lcSSc skin fibroblasts of patients with ACAs. We performed IF to visualize CENPB (green) and the expression of the MHC class II molecules DRB1 (beta 1 chain: purple) and DRB5 (beta 5 chain: red) in SSc and healthy skin fibroblasts. Nuclei were counterstained with DAPI (Blue). Shown are representative pictures of at least 10 micrographs. Arrows indicate colocalization of cytoplasmic/membrane CENPB and both MHCII regions in lcSSc patients (025 and 111), who have ACAs. No colocalization was visualized in dcSSc skin fibroblasts (043 and 116). The scale bar is shown at the bottom right.

To study whether cytoplasmic centromere proteins are responsible for ACA production, we investigated if CENP proteins colocalized with the MHC class II molecules DRB1 and DRB5. We observed colocalization of both DRB1 and DRB5 with CENPB in the cytoplasm/membranes of fibroblasts from two lcSSc patients who have ACAs. We did not observe DRB1 or DRB5 colocalization with CENPB in dcSSc or healthy fibroblasts (Fig. 3 and Supplementary Fig. 7). These data indicate that presentation of CENP proteins by MHC class II molecules in lcSSc skin fibroblasts could initiate an ACA response.

**Fig. 4:**
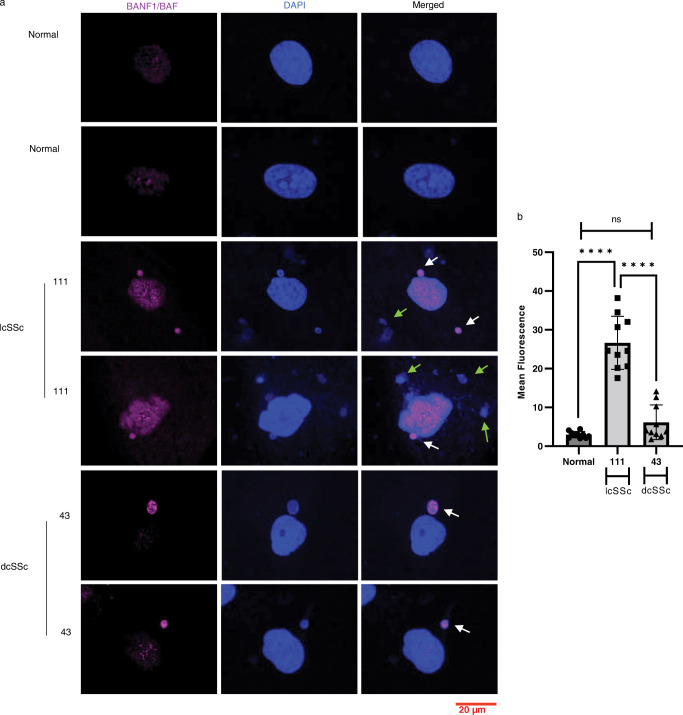
Nuclei and micronuclei membrane integrity in SSc fibroblasts. **a** We performed IF to visualize the expression of BANF1/BAF (purple), a marker for nuclear membrane integrity, in SSc skin fibroblasts. Nuclei and micronuclei were counterstained with DAPI (blue). In contrast to healthy and dcSSc skin fibroblasts (patient 43), abnormal expression of BANF1/BAF was found in the nuclei of the fibroblasts from an lcSSc patient (111) with centromere protein leaking suggesting nuclear membrane disruption. The micrographs also show that the fibroblasts from both of the SSc patients have several micronuclei stained with BANF1/BAF (white arrows) as well as micronuclei without BANF1/BAF staining (green arrows). The scale bar is shown at the bottom right. **b** The bar graph shows the mean fluorescence level of BANF1/BAF in SSc patients compared to healthy skin fibroblasts (*n* = 10 micrographs). Data were analyzed using one-way ANOVA and Tukey’s multiple comparisons test. ****p < 0.0001 ns = *p* 0.305. Data are presented as mean values +/− SD. Source Data are provided as a Source Data file.

We also investigated whether rupture of the nuclear membrane could be responsible for the leaking of centromere proteins. We stained SSc fibroblasts with an anti-BANF1/BAF antibody, a marker for nuclear membrane integrity that accumulates in the nucleus to facilitate nuclear membrane repair^44,45^. In contrast to healthy skin fibroblasts and fibroblasts from a patient with dcSSc, we found increased BANF1/BAF staining in the nuclei of an lcSSc patient that exhibit centromere protein leaking and an ACA response (Fig. 4), The increased accumulation of BANF1/BAF suggests that nuclear membrane disruption might explain, in part, the leaking of proteins into the cytoplasm. We also observed staining of BANF1/BAF in several micronuclei from an lcSSc patient and a dcSSc patient, suggesting nuclear membrane rupture in some of the micronuclei (Fig. 4).

### γ-H2AX protein localizes to micronuclei

To add further context to centromere DNA damage in SSc, we measured the expression of γ-H2AX in SSc fibroblasts. We observed low or negligible staining of γ-H2AX in nuclei of cells that do not show CIN. However, we observed increased γ-H2AX in micronuclei of SSc fibroblasts compared to normal fibroblasts (Supplementary Fig. 8). Strikingly, γ-H2AX also colocalized with CENPB on micronuclei and/or cytoplasmic DNA, indicating that SSc fibroblasts have damaged centromere DNA sequences.

### Increased expression of CENPA and cGAS-STING in SSc

**Fig. 5:**
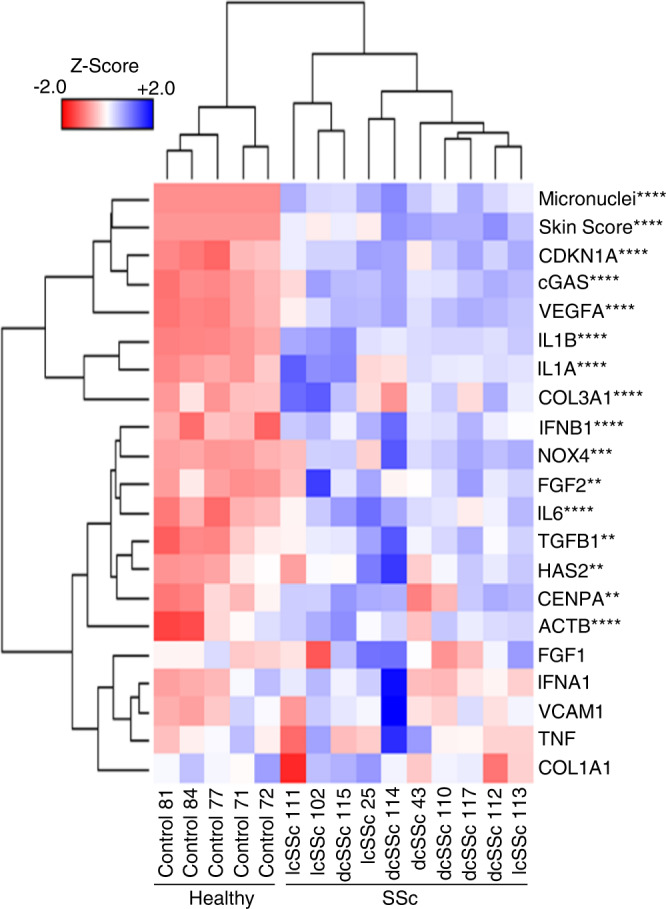
Expression of profibrotic, proinflammatory, vasculopathy, ROS, and cGAS-STING genes in fibroblasts of SSc patients. A heatmap analysis depicting gene expression content (rows) obtained by qRT-PCR in 50 ng of RNA from skin fibroblasts dissected from healthy individuals and patients with lcSSc and dcSSc. Unsupervised hierarchical clustering analysis was conducted to ascertain if distinct genetic signatures separate healthy fibroblasts from lesion fibroblasts. Normal samples (denoted as “control”) do not cluster with diseased samples, suggesting that gene expression alterations in fibroblasts obtained from SSc patients diverge from variations that occur at baseline in fibroblasts obtained from healthy individuals. The number of micronuclei found per 500 nuclei count and the mRSS are included in the analysis. Data obtained in three replicates were analyzed using a multiple unpaired *t*-test, assuming a gaussian distribution. Stars indicate statistically significant differences between the control cells and SSc patients in a *t*-test analysis (**p < 0.1, ***p < 0.001, ****p < 0.0001). Source Data are provided as a Source Data file.

Having observed genetic and epigenetic centromeric alterations in fibroblasts from skin lesions of SSc patients, we used qRT-PCR to ascertain whether the expression of the centromere-defining epigenetic factor CENPA is affected. In contrast to healthy fibroblasts, we found significantly increased levels of *CENPA* in fibroblasts from all lcSSc and 4 of 6 dcSSc patients (Fig. 5, Supplementary Fig. 9). We further measured the levels of fibrotic/inflammatory genes and found that *IL6*, *IL1A, IL1B*, *COL3A1*, and *TGFB1* were all significantly overexpressed in lcSSc and dcSSc skin fibroblasts (Fig. 5, Supplementary Fig. 9). Further, a significantly increased abundance of *FGF2* was seen in dcSSc fibroblasts as well as in some but not all lcSSc patients (Supplementary Fig. 9). We did not find significant changes in the transcript levels of *FGF1*, *COL1A1, TNF* or the housekeeping gene *GAPDH* used for normalization (Supplementary Fig. 9). Given that ROS could produce centromere alterations and CIN as well as modulate TGFB1 activation and fibrosis in SSc^8^, we measured the levels of ROS-derived genes, finding significant overexpression of *CDKN1A* and *NOX4* in SSc fibroblasts (Fig. 5, Supplementary Fig. 9). Finally, having observed micronuclei in all SSc patients we reasoned that cellular surveillance of cytoplasmic DNA could trigger the activation of the cGAS-STING pathway, which is involved in innate cellular immunity and linked to autoimmunity^42,43^. Expression levels of *cGAS*, and its downstream targets *IFNB1*, and *IL6*, but not *IFNA1*, were significantly upregulated in SSc fibroblasts, suggesting activation of the cGAS-STING pathway in SSc (Fig. 5, Supplementary Fig. 9).

### Correlation analysis of centromere DNA and RNA transcripts

We compared the levels of gene expression in healthy (*n* = 5) and SSc patients (*n* = 10) with centromere DNA changes and CIN variables (micronuclei), to identify possible correlations that may have clinical impact. A heatmap analysis indicated that a strong correlation exists between the expression of genes involved in SSc fibrosis, CENPA, ROS, cGAS-STING, and micronuclei, suggesting that these variables are interconnected (Supplementary Figs. 10 and 11). Another strong correlation arises between having centromere deletions in several chromosomes (Supplementary Fig. 11). Interestingly an inverse correlation exists between centromere deletions in chromosomes 5, 8, and 21 and increased expression of fibrotic genes IL1A, IL1B, and IL6. Contraction of centromere 21 and expansion of centromere 12 correlated with the expression of ROS genes (*NOX4* and *CDKN1A*), as well as overexpression of *cGAS*, *VEGFA*, *TGFB1*, and *FGF2* (Supplementary Fig. 11). The analysis of gene expression values shows strong correlation between the fibrotic genes *COL3A1*, *IL1A*, and *IL1B* (*r* = 0.77 to 0.825), between micronuclei formation and activation of *cGAS* (*r* = 0.6095) and *IFNB1* (*r* = 0.9562), between activation of the ROS genes *NOX4* and *CDKN1A*, and between the SSc pro-fibrotic gene *TGFB1* (*r* = 0.846 and 0.954, respectively) (Supplementary Figs. 10 and 11).

A positive correlation was found between *CENPA* levels and activation of several fibrotic genes [*IL6* (*r* = 0.7065)*, FGF1* (*r* = 0.5544)*, IL1B* (*r* = 0.6094), and *TGFB1* (*r* = 0.5755)] as well as with *CDKN1A* (*r* = 0.7321)*, VEGFA* (*r* = 0.7196)*, cGAS* (*r* = 0.7563), and *IFNB1* (*r* = 0.5223) in all SSc patients tested (See r and *p* values in Supplementary Fig. 12). These data suggest that expression of CENPA is increased in fibroblasts from SSc skin lesions and correlates with the expression of certain profibrotic and proinflammatory markers. The overexpression of CENPA in these abnormally proliferating fibroblasts is strikingly similar to what we and others have observed in cancerous cells, which likewise have centromeric and chromosomal instability^46,47^.

The extent of micronuclei formation strongly correlated with ROS markers *CDKN1A* (*r* = 0.7413) and *NOX4* (*r* = 0.7626), supporting the link observed between ROS and development of CIN^48^ (Supplementary Fig. 13). Likewise, micronuclei formation strongly correlated with the activation of the cGAS-STING pathway [*cGAS* (*r* = 0.6095), *IFNA1* (*r* = 0.7486), *IFNB1* (*r* = 0.9562)*, IL6* (*r* = 0.5675)], supporting the idea that cytosolic surveillance of the abnormally located DNA in SSc activates the cGAS-STING pathway (Supplementary Fig. 13). While the formation of micronuclei did not correlate with the expression of all fibrotic genes, it did correlate with the expression of key fibrotic genes *FGF1* (*r* = 0.5590) and *TGFB1* (*r* = 0.9039) (Supplementary Fig. 13). cGAS activation strongly correlated with ROS markers. Importantly, cGAS expression strongly correlated with the expression of downstream elements of the cGAS-STNG pathway [*IFNB1* (*r* = 0.6838) and *IL6* (*r* = 0.6641)]. While *cGAS* activation did not correlate with the expression of all fibrotic/proinflammatory genes, it did correlate with the expression of *FGF2* (*r* = 0.7070) and, similar to the formation of micronuclei, it correlated with the expression of *TGFB1* (*r* = 0.6836) (Supplementary Fig. 14).

Multiple correlation analysis confirmed and extended the strongest correlations found in the heatmap (Fig. 5 and Supplementary Fig. 15). For example, activation of *NOX4* and *CDKN1A*, markers of ROS production, correlates with micronuclei formation (Multiple *R*^2^ = 0.6117). The stronger correlation was seen between *NOX4* expression and micronuclei formation with a *TGFB1* (Multiple *R*^2^ = 0.8902). Intimate correlations were seen between micronuclei formation and the activation of *cGAS* and the downstream elements *IFNB1* and *IL6* (Multiple *R*^2^ = 0.48-0.49) as well as between the expression of profibrotic genes *COL3A1*, *IL1A*, and *IL1B* (Multiple *R*^2^ = 0.6415). Activation of CENPA appears to contribute positively to cGAS-STING activation (Multiple *R*^2^ = 0.5385), an effect enhanced by *CDKN1A* activation (Multiple *R*^2^ = 0.6706).

### Activation of cGAS-STING/IFN-β pathway in SSc fibroblasts

**Fig. 6:**
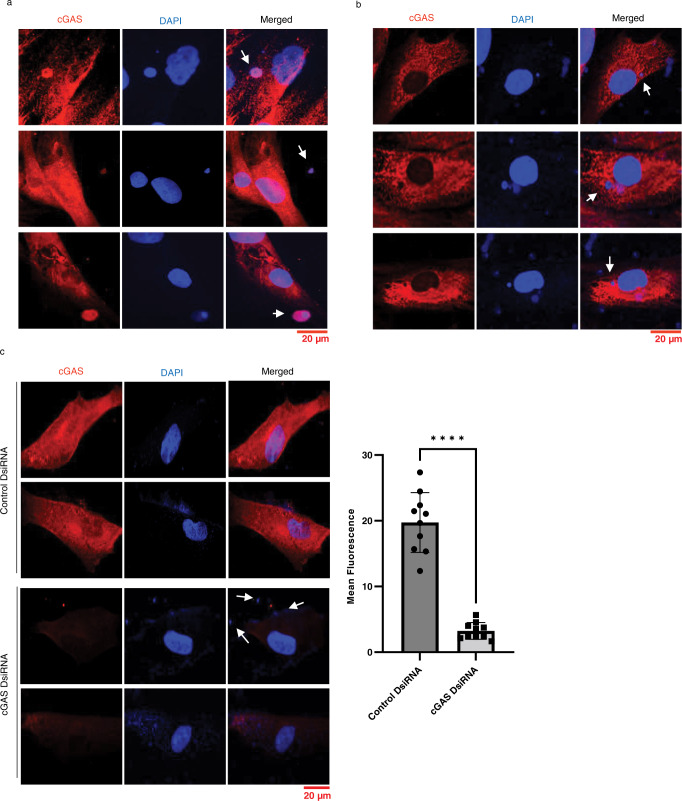
Colocalization of cGAS to micronuclei in SSc fibroblasts. IF analysis of expression of cGAS (red) in SSc fibroblasts. Nuclei and micronuclei were stained in DAPI (blue). **a** Micrographs showing cGAS expression and colocalization to micronuclei in an lcSSc patient (111). **b** Micrographs showing only cGAS expression but not co-localization to micronuclei in the same lcSSc patient (111). **c** Confirmation of the specificity of c-GAS staining in **a** and **b**. Representative fluorescence images and quantification using ImageJ showing reduction of cGAS expression in cGAS DsiRNA transfected lcSSc patient fibroblasts (111) compared to control DsiRNA transfected lcSSc patient fibroblasts (111). The scale bars are shown at the bottom right. Arrows indicate micronuclei. The bar graph shows the mean fluorescence level of cGAS expression in Control DsiRNA-treated cells vs cGAS DsiRNA-treated cells (*n* = 10 micrographs per experiment). ****p < 0.0001. Data are presented as mean values +/− SD. Source Data are provided as a Source Data file.

**Fig. 7:**
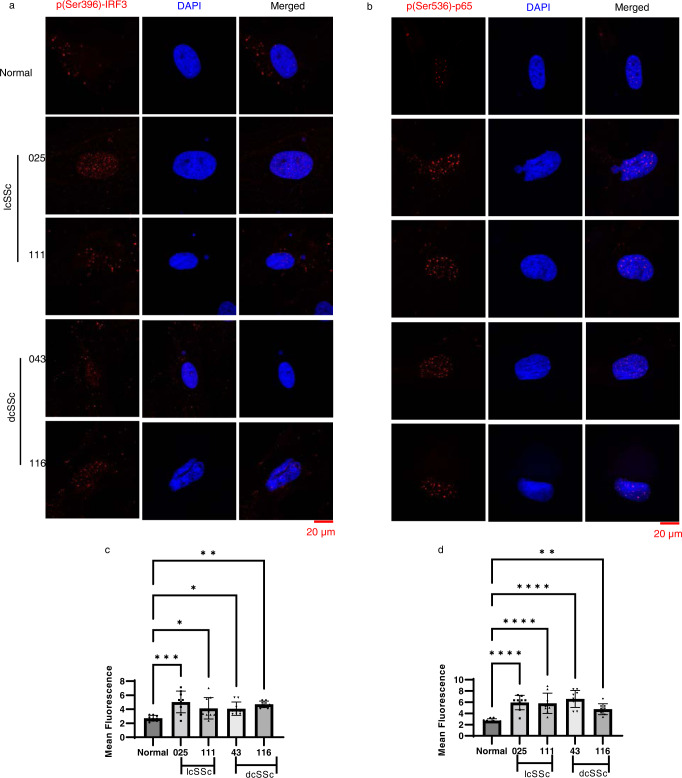
Expression of IRF3 and p65 in SSc skin fibroblasts. IF showing the expression cGAS-STING downstream factors (red) Phospho-IRF3 (**a**) and Phospho p65 (RELA) (**b**) in SSc skin fibroblasts. Nuclei were counterstained with DAPI (blue). IRF3 and p65 are phosphorylated at serine residues following cGAS-STING activation. Phosphorylated IRF3 forms a dimer and translocates to the nucleus to activate IFN-β and other cytokines. P65 (RELA) translocates to the nucleus after cGAS-STING activation. Both proteins activate the IFN-β pathway. The scale bar is shown at the bottom right. The bar graphs (**c**–**d**) show the mean fluorescence level of respective phopho-protein expression in SSc patients compared to healthy skin fibroblasts (*n* = 9 micrographs). Data were analyzed using one-way ANOVA and Dunnett’s multiple comparisons test. *p < 0.05, **p < 0.01, ***p < 0.001, ****p < 0.0001. **a** Normal vs. 025 *p* = 0.0002; Normal vs. 111 *p* = 0.0315; Normal vs. 43 *p* = 0.0406; Normal vs. 116 *p* = 0.0016. **b** Normal vs. 116 *p* = 0.0066), the other *p* values are <0.0001. Data are presented as mean values +/− SD. Source Data are provided as a Source Data file.

Having observed increased micronuclei formation that correlated strongly with the expression of cGAS, IFN-β, and other proinflammatory genes, we investigated whether the cGAS-STING pathway is in fact activated in SSc fibroblasts. We studied whether cGAS protein is increased and colocalized to micronuclei using IF. In agreement with our previous RT-PCR data, we found increased expression of cGAS in lcSSc and dcSSc patients compared to healthy fibroblasts (Fig. 6). While cGAS was detected throughout the cytosol, it accumulated specifically inside and around micronuclei (Fig. 6a, b and Supplementary Fig. 16), suggesting that micronuclei activate cGAS in SSc. Depletion of cGAS using DsiRNA confirmed the specificity of the cGAS staining (Fig. 6c). Next, we evaluated the expression of cGAS-STING downstream factors. We observed increased expression and nuclear translocation of IRF3, the active Ser-396 phosphorylated form, in lcSSc and dcSSc fibroblasts (Fig. 7a). Further, we observed increased Phospho p65 (RELA) expression in the nucleus of both lcSSc and dcSSc fibroblasts (Fig. 7b).

**Fig. 8:**
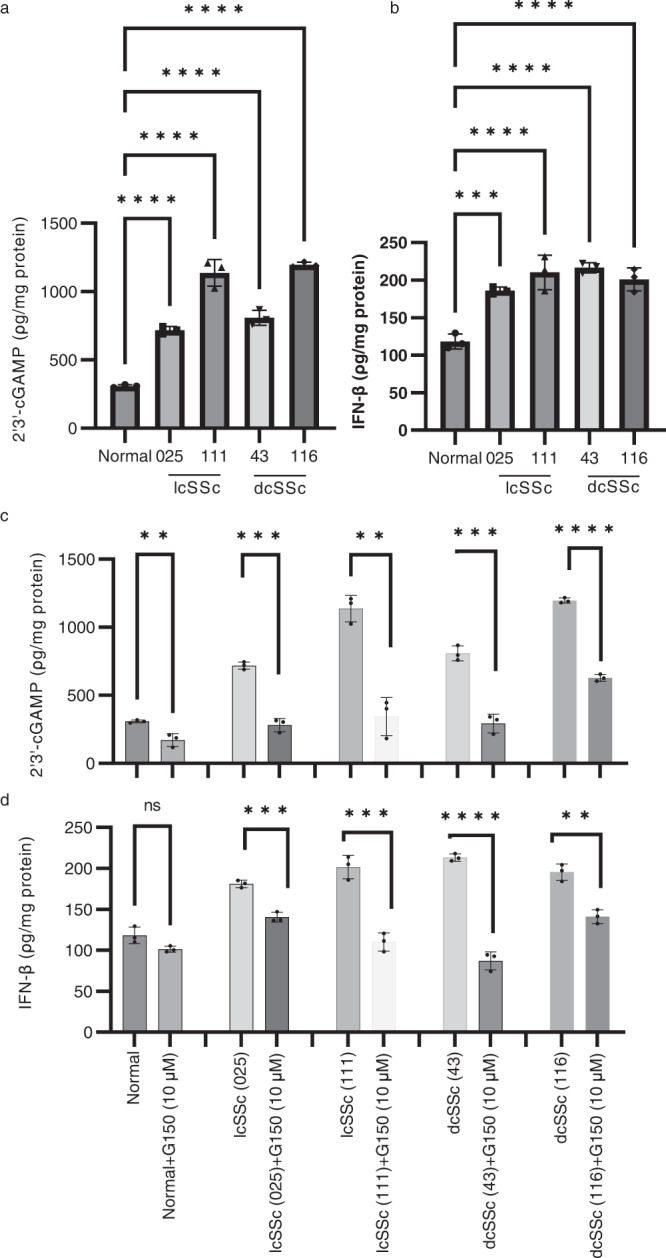
Expression of 2′3′-cGAMP and IFN-β in SSc skin fibroblasts and inhibition of cGAS-STING/IFN-β activity with a cGAS inhibitor. ELISAs were performed to measure the levels of expression of 2′3′-cGAMP (**a**) and IFN-β (**b**) in cell lysates and supernatants, respectively, in cultured skin fibroblasts from healthy,lcSSc (025 and 111), and dcSSc (43 and 116) individuals. Inhibition of the cGAS-STING/IFN-β pathway was assessed using the cGAS specific inhibitor G150. After treatment with G150 (10 µM) for 2 h cells were cultured for additional 24 h and the levels of of expression of 2′3′-cGAMP (**c**) and IFN-β (**d**) were evaluated using ELISA. Data are presented as mean values +/− SD (*n* = 3 independent experiments). Data were analyzed using one-way ANOVA and Dunnett’s multiple comparisons test. *p < 0.05, **p < 0.01, ***p < 0.001, ****p < 0.0001. The exact *p* values can be found in the Source Data file. Source Data are provided as a Source Data file.

Finally, we used ELISA to measure 2′3′ cGAMP, a second messenger directly produced by cGAS after sensing cytosolic DNA, as well as secreted IFN-β. We observed increased production of 2’3’ cGAMP from cell lysates in both lcSSc and dcSSc fibroblasts when compared to healthy fibroblasts (Fig. 8a). We also found that IFN-β was increased in the cell supernatant of lcSSc and dcSSc fibroblasts (Fig. 8b). To confirm that cGAS activation is responsible for the IFN-β production in these cells, we treated the fibroblasts with G150, a cGAS specific inhibitor. We observed that treatment of SSc fibroblasts with G150 strongly reduced the cGAS-induced enzymatic (2′3′ cGAMP) activity and the production of IFN-β in SSc fibroblasts (Fig. 8c, d). Taken together, these data support the idea that the formation of micronuclei contributes to the activation of cGAS-STING and the production of IFN-β in SSc fibroblasts.

## Discussion

**Fig. 9:**
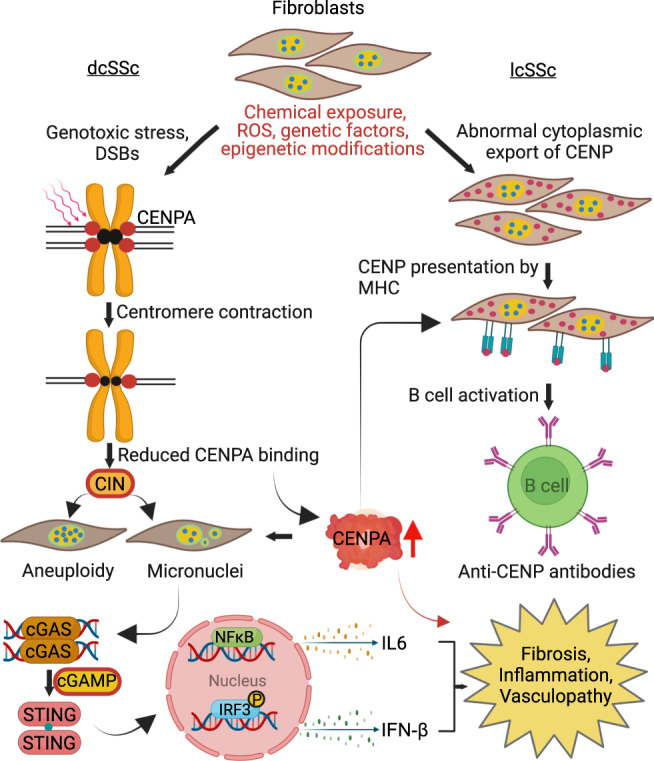
The model depicting how centromere alterations and chromosome instability could impact the etiology of Systemic Sclerosis. The damage to skin fibroblasts leads to centromere DNA alterations and/or abnormal centromere protein deposition in the cytoplasm. In dcSSc, centromere damage produces centromeric contractions and reduction of CENPA deposition at the centromere, leading to unstable kinetochore and microtubule attachment. These processes result in chromosome instability (CIN), characterized by aneuploidy and micronuclei formation and upregulation of CENPA. The overexpression of and/or mislocalization of CENPA alters gene expression as we have seen in cancer^45^. In lcSSc, altered deposition of CENP proteins leads to CIN characterized by micronuclei formation as well as to excess of CENPA deposition. In lcSSc patients who develop ACAs, centromere DNA is somewhat affected and centromere proteins leak into the cytoplasm, further increasing the level of CENPA expression, which further drives CIN characterized by micronuclei formation. Given that fibroblasts from patients with SSc are known to express MHC on their surface, these MHC molecules likely present the cytoplasmic CENPs to B cells to induce production of ACAs. The presence of extranuclear DNA (micronuclei) in all SSc fibroblasts leads to activation of the cGAS-STING/IFN-β pathway and autoimmunity. DSBs doubled-strand breaks, IFN-β interferon beta, IL6 interleukin 6, CENP centromere protein, MHC major histocompatibility complex.

In the present paper we demonstrate that skin fibroblasts from SSc patients, the cellular drivers of skin fibrosis, show genetic and epigenetic centromere alterations, as well as CIN. ROS appear to contribute to centromere DNA damage leading to CIN in the form of aneuploidy and/or micronuclei. Strikingly, our data indicates that micronuclei formation in SSc is intimately linked to the activation of the cGAS-STING pathway. Our data also indicate that centromere alterations are to linked to clinical manifestations of skin fibrosis. These findings provide the framework for a model in which centromere abnormalities and CIN are central to the molecular processes that drive autoimmunity in SSc (Fig. 9). In addition, our model proposes that cytoplasmic leakage of nuclear centromere proteins leads to production of ACAs in lcSSc patients, especially as MHC Class II molecules in skin fibroblasts associate with centromere proteins (see discussion below) (Fig. 9).

Over the years, several groups noted an abundance of chromosomal abnormalities in SSc^35–40^, including chromatid gaps and breaks, acentric fragments, dicentric and ring forms. Polyploidization, endoreduplication, and fragmentation of chromosomes were also noted. These aberrations were specific to lymphocytes, lesion fibroblasts, and bone marrow cells. These cytogenetic abnormalities were more common in advanced forms of SSc, but not in milder forms like lcSSc, which is in agreement with our findings. However, we did not observe cytogenetic abnormalities or segregation defects in dcSSc or lcSSc lymphocytes; the reasons for these discrepancies remain to be clarified. In our study, centromere defects, cytogenetic abnormalities, and segregation defects were limited to lesion fibroblasts of SSc patients. It is possible that a fibrotic milieu rich in DNA-damaging reactive oxygen species plays a causal role in the centromere defects we observed in SSc lesion fibroblasts. Indeed, our γ-H2AX IF data indicates that SSc fibroblasts have elevated numbers of micronuclei with centromere-specific DNA damage.

Interestingly, we did not find aneuploidy in lcSSc lesion fibroblasts, despite the presence of micronuclei. A previous report showed that lagging chromosomes entrapped in micronuclei are more often entrapped in the correct daughter cell^49^. Another report has demonstrated that aneuploid cells are cell-cycle arrested and eliminated by the immune system^50^. The elevated aneuploidy we observed in dcSSc fibroblasts suggests that some aneuploid fibroblasts avoid cell-cycle arrest and escape immune clearance in dcSSc, but not lcSSc. One explanation is that immunosuppressive therapy may play a role in allowing aneuploid cells to avoid cell-cycle arrest. However, 2 dcSSc patients showed aneuploidy in skin lesion fibroblasts without ever receiving immunosuppressive therapy. Rather, we propose that the centromere genetic deletions in dcSSc contribute to increased aneuploidy and may account for some of the differences between it and the generally less aggressive lcSSc. The centromere genetic deletions in dcSSc fibroblasts are similar to the alterations we have previously observed in cancer cells^34^. Interestingly, patients with SSc are known to have an increased risk of cancer^1–4^, with dcSSc showing higher incidence than lcSSc^51^. Indeed, dcSSc fibroblasts behave much like cancer cells in the sense that they can migrate and differentiate abnormally within the skin and visceral organs.

Our study fully describes marked centromeric abnormalities in SSc patients. From the epigenetic point of view, it was striking that the centromere proteins CENPA and CENPB, which are found almost exclusively in the nucleus, colocalized in the cytoplasm of fibroblasts from 50% of the patients with lcSSc, but not dcSSc. The leaking of CENPA and CENPB into the cytoplasm correlated 100% with the presence of ACAs in patient blood and the CENP protein aggregates colocalized with MHC class II molecules. It is quite possible then that skin fibroblasts from these patients can present cytoplasmic centromere antigens to B-cells and trigger ACA production^52–54^ (Fig. 9). ACAs were discovered in patients with a subset of lcSSc, previously called CREST. ACAs recognize a range of centromere proteins but mostly react to CENPA and CENPB^6^. Clinically, these antibodies correlate with lcSSc, lower skin scores, and a higher rate of pulmonary arterial hypertension^14^. The fact that both CENPA and CENPB and MHC class II molecules cluster in the cytoplasm or membrane of lcSSc lesion fibroblasts from patients that developed ACAs suggests that centromere structures are somehow ejected from the nucleus to the cytoplasm and elicit an immune response. It is possible that a similar mechanism of “centromere leaking” and presentation occurs in other diseases that elicit ACA responses, such as primary biliary cholangitis and overlap connective tissue disease Sjogren’s syndrome^55^.

Overexpression and/or mislocalization of CENPA, which also correlate with micronuclei formation in our study, may have an important role in the development of CIN in SSc. In fact, a recent study shows a molecular link between CENPA overexpression and aneuploidy^56^. Shrestha et al. determined that CENPA overexpression leads to a reduction in kinetochore protein localization and kinetochore assembly, ultimately leading to CIN in the form of lagging chromosomes and micronuclei^56^. Therefore, we propose that elevated CENPA expression and/or mislocalization contributes to CIN in both forms of SSc.

Importantly, our correlation studies indicate that the formation of micronuclei in SSc is linked to the cGAS-STING pathway. It has been shown that sensing of cytosolic DNA by cGAS activates an immune pathway to eliminate cells containing cytosolic DNA^41,42,57–59^. Further, cGAS-STING activation has been linked to autoimmunity^42,57^. Our study indicates that formation of micronuclei may trigger the autoimmune component in SSc patients by activating the cGAS-STING pathway. This observation highlights the plausibility of using cGAS-STING inhibitory therapies in the treatment of SSc^59,60^, a very important issue as presently there are only very limited and usually non-specific therapeutic options available for this often-devastating disease.

In summary, we find that, in addition to the autoimmunity and vasculopathy component, the pathology of SSc involves centromere genetic and epigenetic alterations that lead to chromosome missegregation. ROS may produce breaks and/or interfere with the repair of centromere sequences, altering the deposition of centromeric proteins on centromere sequences, ultimately leading to CIN. Malfunction and overexpression of CENPA appear to contribute to chromosome missegregation and alter the expression of fibrotic genes in SSc. Finally, our data suggest that CIN in fibroblasts of SSc patients activates the cGAS-STING pathway, thus linking the centromeric and chromosomal defects in SSc to the autoimmune component of the disease. Our results suggest that targeting cGAS-STING, a pathway for which agents are presently being developed to treat autoimmune diseases and cancer, may prove to be a viable treatment option for patients with SSc.

## Methods

### Study approval

The University of Michigan Medical Institutional Review Board (HUM00065044) approved the procedures described in this study and patients were recruited upon informed written consent. The study design and conduct complied with all relevant regulations regarding the use of human study participants and was conducted in accordance with the criteria set by the Declaration of Helsinki. A description of the human research participants and clinical data used in this study is shown in Supplementary Table 1. Data are de-identified to the investigators.

### Human research participants, skin biopsies, blood collection, and cell culture

Patients fulfilled 2013 ACR/EULAR criteria for SSc^61^ and were categorized as lcSSc and dcSSc based on Leroy criteria^62^. The demographics and clinical data of the SSc patients are shown in Supplementary Table 1. Skin biopsies were collected through two 4 mm punch biopsies from the mid outer forearm 3 to 4 inches from the ulnar styloid. Modified Rodnan skin scores ranged from 2 to 14+ for lcSSc biopsies, except one that had a score of 0. Modified Rodnan skin scores ranged from 7 to 34+ in dcSSc biopsies.

Fibroblasts were isolated and separated from endothelial cells in skin biopsies obtained as previously described^63^. Briefly, the dermis biopsy was digested with 0.2% collagenase and grown in RPMI medium (Lonza) medium supplemented with 10% FBS, 400 U/ml penicillin and 50 µg/ml streptomycin. After digestion, endothelial cells were labeled with magnetic anti-CD31 antibody (CD31 MicroBead Kit) and added to LS columns (Miltenyi Biotech) fastened to MidiMACS separator. Negatively selected cells, which were fibroblasts, were cultured in RPMI medium (Lonza) supplemented with 10% FBS and antibiotics. The experiments were carried out on fresh fibroblasts between passages 1 and 10.

Blood was collected by venipuncture and the peripheral blood mononuclear cells (PBMCs) were separated using a Histopaque 1077 (Sigma) density gradient centrifuging at 400 ×  *g* for 30 min. PBMCs were washed three times with a phosphate buffer saline (PBS) solution and grown in 10 cm dishes in DMEM medium (Gibco) supplemented with 10% human serum (Sigma) and antibiotics for 2 h. Adherent cells (monocytes) were separated from suspension cells (lymphocytes). Monocytes were washed five times with PBS in order to remove contaminant lymphocytes and then cultured with DMEM medium supplemented with 10% human sera for 7 days until they differentiate into macrophages. Lymphocytes were washed twice with PBS and cultured in RPMI medium supplemented with 10% FBS, penicillin-streptomycin, and activated with 5 μg/ml phytohemagglutinin (PHA) for 7 days.

### Antibodies

Primary Antibodies: anti-CENPA (MBL international 1:500, Code # D115-3), anti-CENPB primary antibody (Santa Cruz Biotechnology, CENPB Antibody (C-10) 1:50: sc-376392, pSer139-γ-H2AX antibody (Cell Signaling 1:400, Cat # 9718), BANF1/BAF antibody (abcam,1:100, EPR7668), MHCII DRB1 antibody (Abclonal, 1:100, Cat # A7685), Lamin B1 antibody (Proteintech, 1:1000, Cat # 66095-1-lg), cGAS antibody (Abclonal, 1:100, Cat# A8335), p(Ser396)-IRF antibody (Cell Signaling, 1:100, Cat # 29047), p(Ser536)-p65 antibody (Abclonal, 1:100, Cat # AP0123), MHCII DRB5 antibody (Abclonal, 1:100, Cat # A12726), anti-GAPDH antibody (Abcam, ab 9484), anti-Histone H3 (tri methyl K9) antibody (Abcam, ab8898), anti-CENPA antibody [3-19] (Abcam, ab13939).

Secondary Antibodies: Alexa Flour 594 rabbit anti-mouse IgG 1:1000, Thermo Fischer Scientific, Cat No A27027, Alexa Flour 488 rabbit anti-mouse IgG 1:1000, Thermo Fischer Scientific, Cat No A27023. Alexa fluor 647 Goat anti-rabbit IgG, ThermoFisher, 1:1000, Cat # A32733TR

### DNA and RNA isolation

DNA extraction was performed on primary cell lines using the DNeasy Blood and Tissue Kit (Qiagen) according to manufacturer’s instructions. RNA was removed by treating the lysates in column with the DNAse-free RNAse set (Qiagen). DNA was stored at −20 °C. RNA was extracted using the RNeasy mini kit (Qiagen) according to the recommendations described by the manufacturer and treated with RNAse free DNase (Roche). DNA and RNA concentrations were measured using the Qubit 4 fluorometer (Thermo Fischer Scientific).

### Rapid centromere target PCR assay

To measure the size of the centromeres, PCR was performed on DNA samples from primary cell lines. Briefly, the copy numbers for each centromere array, the centromeric proviruses K111 and or K222, and single-copy genes were measured by qPCR using specific primers and PCR conditions as previously reported^33,34^. Briefly, the quantitative PCR was carried out using Radiant Green Lo-Rox qPCR master mix (Alkali Scientific) in a final volume of 20 µL with an enzyme activation step of 10 min at 95 °C followed by 16–35 cycles consisting of 15 s of denaturation at 95 °C and 30 s of annealing/extension at the temperatures specified in our previous report^33^. Amplification of D13Z1 and D21Z1 was performed using LNA primers and clamps. The specificity of the qPCR assay detecting the centromere of unique chromosomes was assessed using DNA samples from human/rodent cell hybrids, each one containing a single human chromosome. The relative copy number was calculated in 50 ng of DNA.

### Real time RT-PCR

To measure the RNA expression levels of centromere CENPA, profibrotic and proinflammatory genes, as well as genes involved in ROS, vasculopathy, and cGAS-STING pathway, we conducted qRT-PCR using the SuperScript III Platinum One-Step qRT-PCR Kit with ROX passive reference dye (Thermo Fisher Scientific). cDNA synthesis was performed at 50 °C for 30 min. Taq polymerase was activated for 2 min at 95 °C and the PCR was carried out in 35 cycles consisting of 15 sec of denaturation at 95 °C and 30 s of annealing/extension at 60 °C. The primers used for the RT-PCR studies are listed in Supplemental Table 4.

### Immunofluorescence (IF)

The binding of centromeric proteins CENPA and CENPB at chromosomes/nuclei was visualized by IF. Cells were synchronized in metaphase for 16 h in 10 μg/ml colchicine solution (KaryoMAX® Colcemid™, Thermo Fischer Scientific) in PBS. Cells were detached with trypsin, suspended in 0.56% KCl hypotonic solution for 15 min at 37 °C, and then suspended in hypotonic solution with 0.05% Tween. To make chromosome spreads, cells were spotted onto slides by centrifugation at 290 × *g* for 5 min using a cytospin centrifuge. IF was done by fixing the chromosome spreads with 4% paraformaldehyde (PFA) for 10 min. The chromosome spreads were then washed with PBS, permeabilized two times in PBST (PBS + 0.05% Triton X) for 5 min, and incubated with blocking solution (PBST + 5% Bovine Serum Albumin (BSA)) for 30 min. Chromosome spreads were incubated with mouse anti-CENPA primary antibody (MBL international 1:500, Code # D115-3) for 1 h, washed three times in PBST for 5 min, and then incubated with a red-fluorescent secondary antibody (Alexa Flour 594 rabbit anti-mouse IgG 1:1000, Thermo Fischer Scientific, Cat No A27027) for 1 h. The spreads were washed three times with PBS and then incubated with an anti-CENPB primary antibody (Santa Cruz Biotechnology, CENPB Antibody (C-10) 1:50: sc-376392) for 1 h, washed three times in PBST for 5 min, and then incubated with a green-fluorescent secondary antibody (Alexa Flour 488 rabbit anti-mouse IgG 1:1000, Thermo Fischer Scientific, Cat No A27023) for 1 h. Spreads were washed three times with PBST, and then twice times with PBS. The chromosome spreads were then counterstained with DAPI solution (ProLong® Gold Antifade Mountant with DAPI, Thermo Fischer Scientific) and visualized in a NIKON Eclipse Ti-S Inverted fluorescent microscope. Quantitation of fluorescent images was performed with ImageJ.

For the detection of γ-H2AX, BANF1/BAF, Lamin B1, cGAS-STING proteins, and MHC class II molecules, cells were fixed with 4% PFA, permeabilized with PBS-0.2% Tween-20, blocked with 5% goat serum, stained with primary antibody for 1 h, washed three times and stained with the fluorochrome-conjugated secondary antibody for 1 h. pSer139-γ-H2AX antibody (Cell Signaling 1:400, Cat # 9718), BANF1/BAF antibody (abcam,1:100, EPR7668) and MHCII DRB1 antibody (Abclonal, 1:100, Cat # A7685) were visualized with a purple fluorescent secondary antibody (Alexa fluor 647 goat anti-rabbit IgG, ThermoFisher, 1:1000, Cat # A32733TR). Lamin B1 antibody (Proteintech, 1:1000, Cat # 66095-1-lg) was visualized with a green-secondary antibody (Alexa Flour 488 rabbit anti-mouse IgG 1:1000, Thermo Fischer Scientific, Cat # A27023). cGAS antibody (Abclonal, 1:100, Cat# A8335), p(Ser396)-IRF antibody (Cell Signaling, 1:100, Cat # 29047), p(Ser536)-p65 antibody (Abclonal, 1:100, Cat # AP0123), and MHCII DRB5 antibody (Abclonal, 1:100, Cat # A12726) were visualized with a red-fluorescent secondary antibody (Alexa Flour 594 rabbit anti-mouse IgG 1:1000, Thermo Fischer Scientific, Cat # A27027).

### Transfection of DsiRNA

Dicer-substrate short interfering RNAs (DsiRNA) for use as a negative control (Catalog # 51-01-14-03) and to target cGAS (Duplex name: hs.Ri.MB21D1.13.2) were purchased from Integrated DNA Technologies. DsiRNAs were transfected following a standard protocol using Lipofectamine 3000 (Invitrogen). Briefly, 0.2 × 10^4^ lcSSc fibroblasts were cultured on each well of an 8-well chamber slide using complete growth media. The fibroblasts were transfected with 10 nM DsiRNA. cGAS protein expression was evaluated using immunofluorescence 48 h after transfection.

cGAS DsiRNA sequence:

5′-AAGGUGAAAUAUUAUCAGCUUCUAA-3′

3′-UCUUCCACUUUAUAAUAGUCGAAGAUU-5′

Control DsiRNA sequence:

5′-CGUUAAUCGCGUAUAAUACGCGUAT-3′

3′-AUACGCGUAUUAUACGCGAUUAACGAC-3′

### Cell extract preparation and immunoblot analysis

Cells were pelleted and dissolved in 2% SDS and heated in a boiling water bath for 15 min. Extracts were supplemented with beta-mercaptoethanol and heated for an additional 2 min at 95 °C. Cytoplasmic and nuclear fractions were separated using the NE-PER™ Nuclear and Cytoplasmic Extraction Reagents (Thermo Fischer scientific) following the protocol described by the manufacturer. Samples were separated using SDS/PAGE, transferred to PVDF membranes, and immunoblotted using, anti-beta actin antibody (Abcam, ab227387), anti-GAPDH antibody (Abcam, ab 9484), anti-Histone H3 (tri methyl K9) antibody (Abcam, ab8898), and anti-CENPA antibody [3-19] (Abcam, ab13939), and the respective secondary antibodies.

### ELISA assays for cGAS-STING activation and pharmacological inhibition

Quantification of 2′3′ cGAMP in skin fibroblast lysates was performed according to the manufacturer’s recommendation (Cayman Chemical, Cat # 501700). IFN-β in cell supernatants was quantified using the Human IFN-Beta ELISA kit (Proteintech, Cat # KE00187) according to the manufacturer’s protocol. To confirm cGAS activation in scleroderma, cGAS was inhibited by treating the cells with G150 (10 µM, Cayman Chemicals) for 2 h. G150 is a specific inhibitor of cGAS^59^. G150-treated cells were cultured for additional 24 h in drug-free medium.

### Statistics and reproducibility

The PCR values obtained in the study were normalized by the total amount of DNA as shown in the figure legends. PCR data were obtained in three replicates. Western blot and ELISA quantitation was performed with three independent image reads. Immunofluorescent data were analyzed in 10 micrographs, unless it is indicated otherwise. The Z-scores were calculated by determining the number of standard deviations a copy number value of a given alpha repeat is away from the mean of the values in the same group, assuming a normal distribution. Heatmaps and statistical analyses were generated using GraphPad Prism version 9.0.0 for Windows, and hierarchical clustering analysis was performed using the gplots, RcolorBrewer, and plotrix packages within the R Studio version 1.14.1103. Data were log2 normalized to the median values of all or one of the healthy samples. Spearman correlation heatmaps indicate the R values. 3D scatter plots were generated using R. Spearman and Pearson correlation analysis and multiple correlation R-squared were used to compare significant correlations. Chi square analysis was performed to calculate significant regression models between cytoplasmic CENPA staining and presence of ACAs in blood. Data were analyzed either by student’s *t*-test or by one-way analysis of variance (ANOVA) followed by Tukey or Dunnett’s multiple comparisons test. A probability of *p* < 0.05 was considered statistically significant. Figure 9 was created using BioRender.

### Reporting summary

Further information on research design is available in the Nature Portfolio Reporting Summary linked to this article.

## Supplementary information

Supplementary Information

Reporting Summary

## Data Availability

The data generated in this study are provided in the Source Data file. Source data are provided with this paper.

## Source data

Source Data
