# Supplementary material for: Centromere defects, chromosome instability, and cGAS-STING activation in systemic sclerosis
Source: Nat Commun. 2022 Nov 18;13:7074. doi: 10.1038/s41467-022-34775-8 (PMC9674829; doi:10.1038/s41467-022-34775-8)
Supplement: Supplementary file 1 — Supplementary Information [file 41467_2022_34775_MOESM1_ESM.pdf]

Supplementary Information

Centromere Defects, Chromosome Instability, and cGAS-STING Activation in Systemic Sclerosis

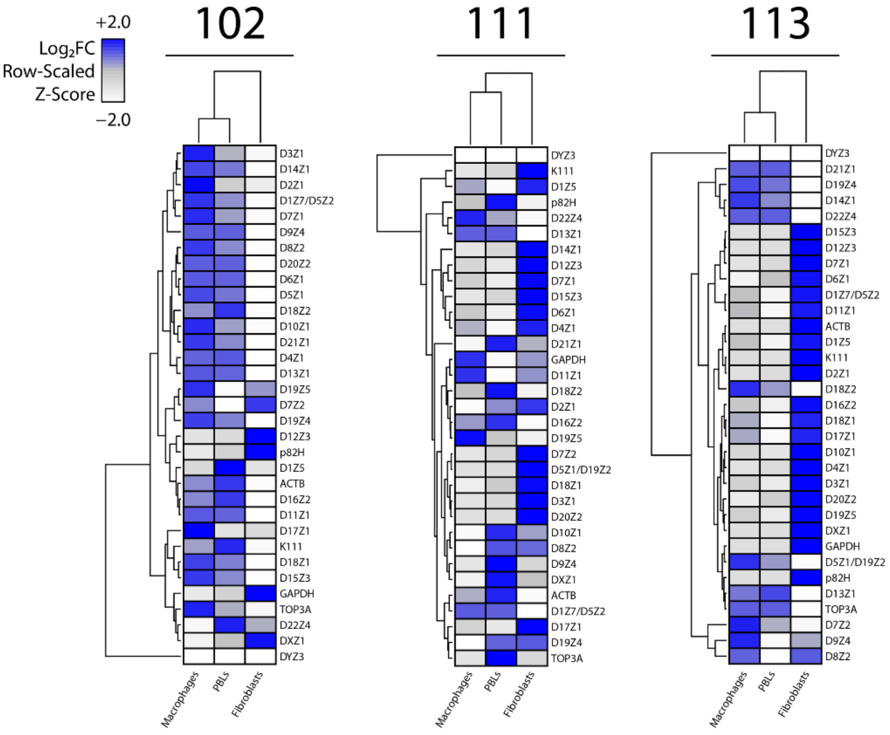

Supplementary Fig. 1. Centromere size variation in lcSSc primary fibroblasts and blood cells. Heatmaps representing the abundance of α-satellites specific for each centromere array (rows) obtained by qPCR of 50 ng of DNA from blood macrophages, peripheral blood lymphocytes (PBLs), and fibroblasts dissected from skin lesions of lcSSc individual patients (columns). The color gradient bar represents relative abundance (left). Unsupervised hierarchical clustering analysis was conducted to ascertain if distinct genetic signatures separate healthy blood cells (macrophages and PBLs) from diseased fibroblasts. Hierarchical clustering shows a closer relationship in the centromere landscape of macrophages and PBLs as compared to affected fibroblast tissue in all three patients. Overall loss or gain of centromeric material could be found in fibroblasts as compared to blood cells. The nomenclature of these α-satellites begins with the letter D, followed by their chromosome number (1–22, X or Y), followed by a Z, and a number indicating the order in which these sequences were first discovered. The DYZ3 repeat accurately represents the gender of the individuals (white: female; blue: male). Source Data are provided as a Source Data file.

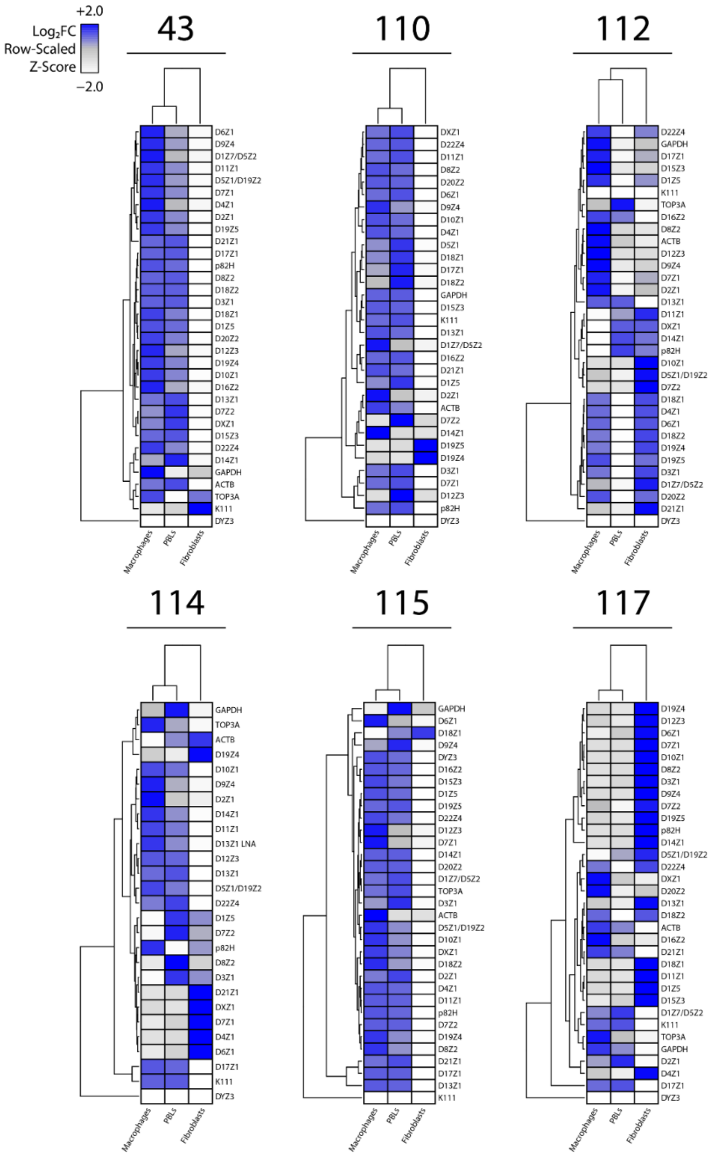

Supplementary Fig. 2. Centromere size variation in dcSSc primary fibroblasts and blood cells. Heatmaps representing the abundance of α-satellites specific for each centromere array (rows) obtained by qPCR of 50 ng of DNA from blood macrophages, peripheral blood lymphocytes (PBLs), and affected fibroblasts dissected from skin lesions of individual dcSSc patients (columns). Unsupervised hierarchical clustering analysis was conducted to ascertain if distinct genetic signatures separate healthy blood cells (macrophages and PBLs) from diseased fibroblasts. The color gradient bar represents relative abundance (left). Hierarchical clustering shows a closer relationship in the centromere landscape of macrophages and PBLs compared to affected fibroblast tissue in patients 43, 110, 114, 115, and 117 (but not 112). Loss of centromeric DNA was the predominant picture. The nomenclature of these α-satellites begins with the letter D, followed by their chromosome number (1–22, X or Y), followed by a Z, and a number indicating the order in which these sequences were discovered. The DYZ3 repeat accurately represents the gender of the individuals (white: female; blue: male). Source Data are provided as a Source Data file.

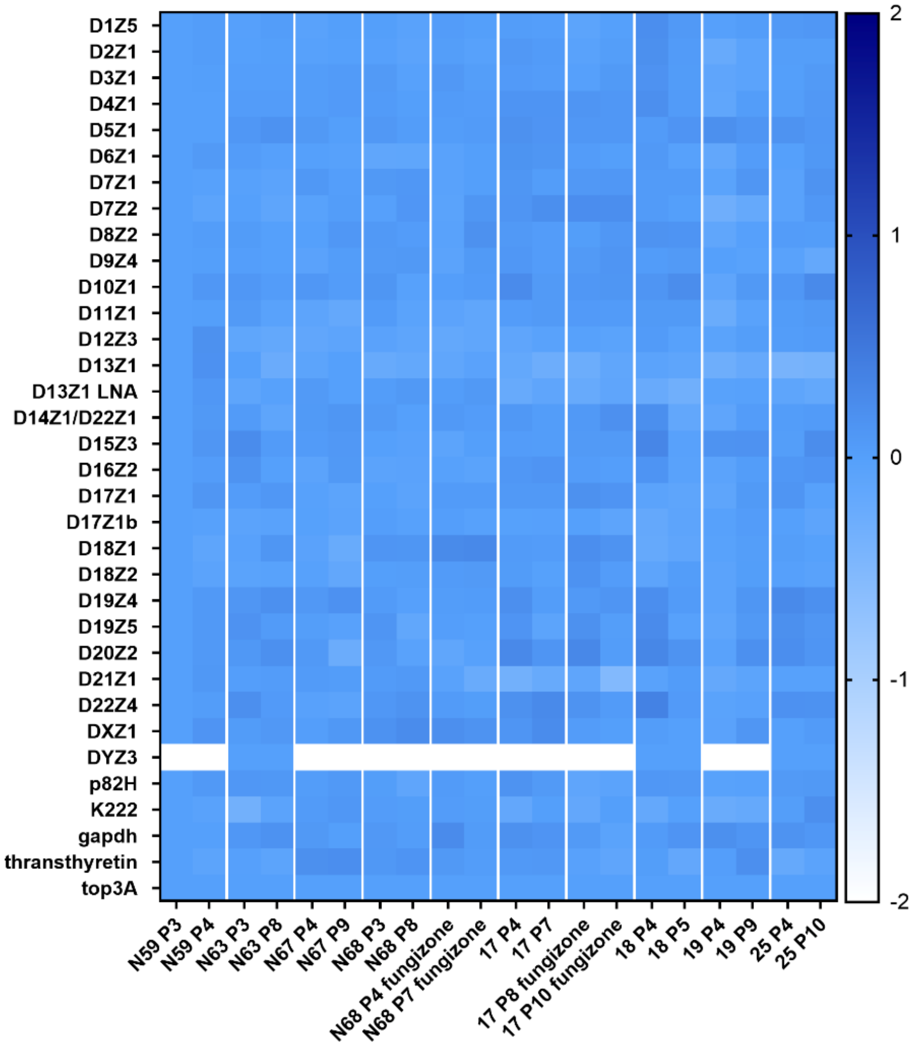

Supplementary Fig. 3. Centromere size does not change with cell passaging or treatment with amphotericin B. A heatmap representing the abundance of α-satellites specific for each centromere array (rows) obtained by qPCR in 50 ng of DNA from fibroblasts dissected from healthy dermis tissue (N59, N63, and N68) and skin lesions of lcSSc patients (17, 18, 19, 25). The passage number is indicated after the letter P as well as whether the cells were further treated with the anti-fungal agent amphotericin B (Fungizone). The color gradient bar represents relative abundance (right) in each centromere array but does not show the variation between arrays. Non-centromeric gene copy numbers (*gapdh*, *transthyretin*, and *top3a*) are also shown. Data depicting α-satellite abundance show log2 differences among each array and the values were normalized to sample N59 P3. No statistical significance was seen between different passage numbers or with amphotericin treatment. The nomenclature of these α-satellites begins with the letter D, followed by their chromosome number (1–22, X or Y), followed by a Z, and a number indicating the order in which these sequences were discovered. The DYZ3 repeat accurately represents the gender of the individuals (white: female; blue: male). Source Data are provided as a Source Data file.

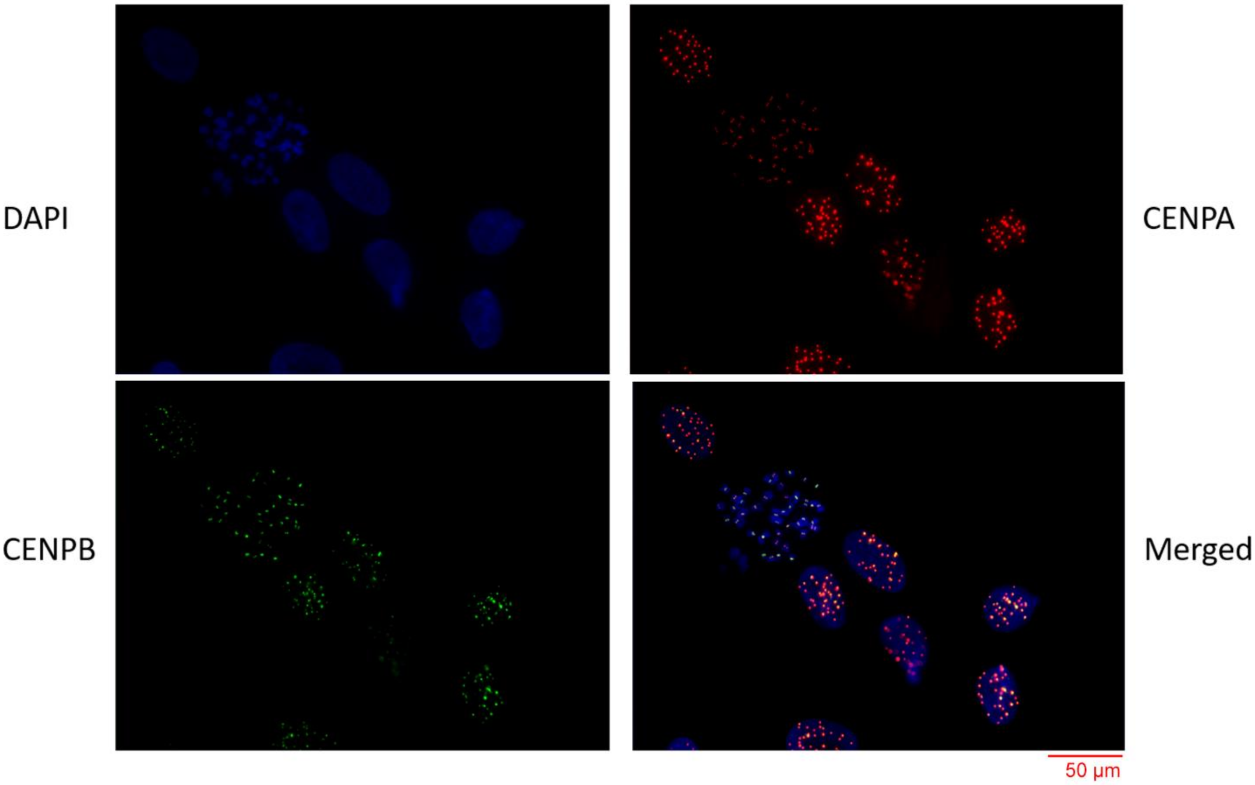

Supplementary Fig. 4. Centromere identity and deposition of centromere proteins in skin fibroblasts from healthy individuals. Fibroblasts isolated from dermal biopsies from healthy individuals were grown in medium with colchicine for 16 h. Nuclei and chromosome spreads were spotted onto slides using a cytocentrifuge. The samples were stained with anti-CENPA (red) and anti-CENPB (green) antibodies and counterstained with DAPI (blue). The pictures illustrate normal deposition and colocalization of centromere proteins CENPA and CENPB in nuclei and chromosomes. Shown is a representation of at least 10 micrographs.

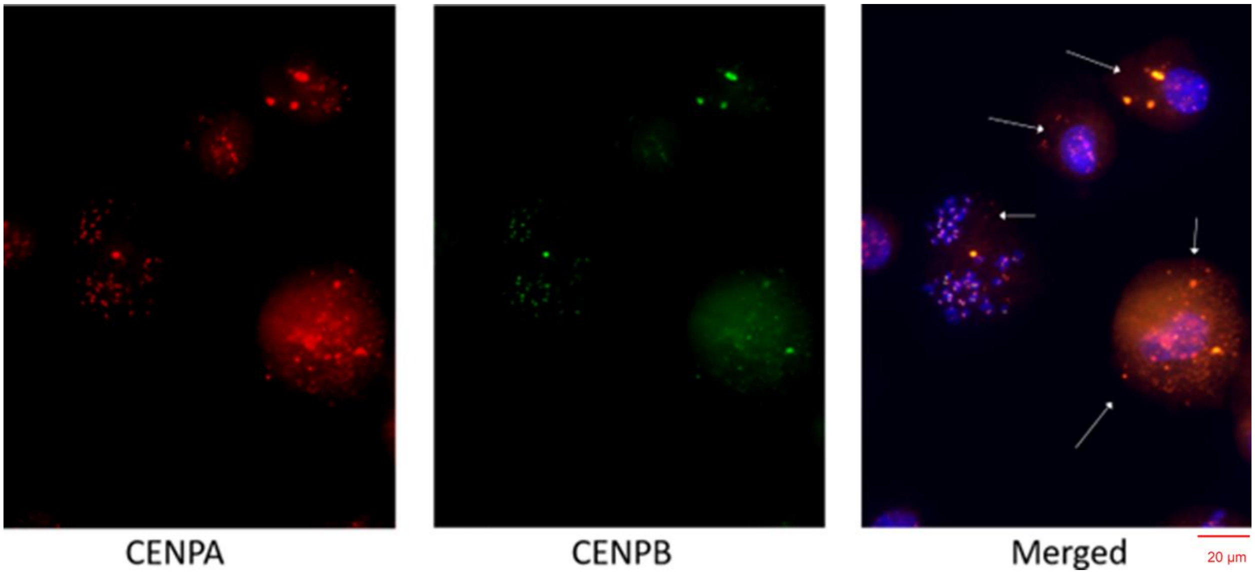

Supplementary Fig. 5. Loss of centromere identity and cytoplasmic mis-localization of centromere proteins in fibroblasts from lcSSc patients with anti-centromere antibodies. Fibroblasts from lcSSc patients were arrested with colchicine for 16 h. Nuclei and chromosomes were spotted onto slides using a cytocentrifuge and stained with anti-CENPA (red) and anti-CENPB (green) antibodies and counterstained with DAPI (blue). Staining and overexpression of both CENPA and CENPB proteins in cytoplasmic regions (arrows) was observed in 6 out of 9 lcSSc patients, who also presented anticentromere antibodies (ACAs) in their blood. A 100% correlation was found between the presence of cytoplasmic staining of CENP proteins and detection of blood ACAs. Shown is a representation of at least 10 micrographs.

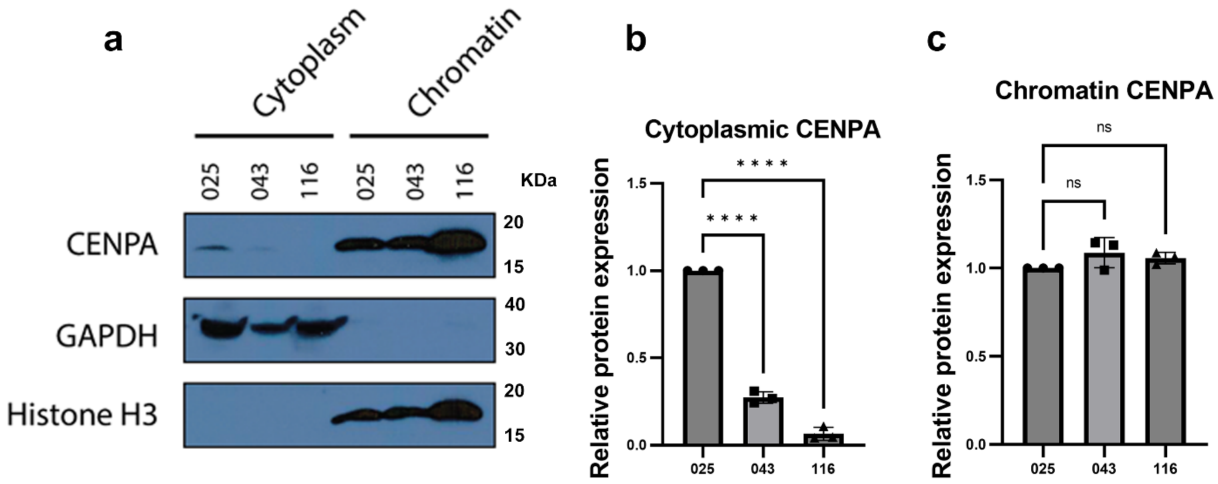

Supplementary Fig. 6. Cytoplasmic and Nuclear CENPA in SSc fibroblasts. a) Western blotting analysis of CENPA, GAPDH and H3K9Me3 in cytoplasmic and chromatin fractions from SSc fibroblasts grown in the presence of colchicine. GAPDH (cytoplasmic) and H3K9Me3 (chromatin) blotting confirmed the specificity and purity of the fractions. CENPA was detected mostly in nuclear chromatin fractions as expected but was found leaked into the cytoplasmic fraction in lcSSc patient 025, who has ACAs. Patients 043 and 116 with dcSSc are seronegative for ACAs. A faint band is seen in the cytoplasmic fraction in patient 043. A quantitative description of cytoplasmic and chromatin CENPA is depicted on b and c. Data were analyzed using one-way ANOVA and Dunnett's multiple comparisons test in three ImageJ readscaptures. **** = $p < 0.0001$, panel c) 025 vs. 043 $p = 0.1496$; 025 vs. 116 $p = 0.3684$. Data are presented as mean values +/- SD. Source Data are provided as a Source Data file.

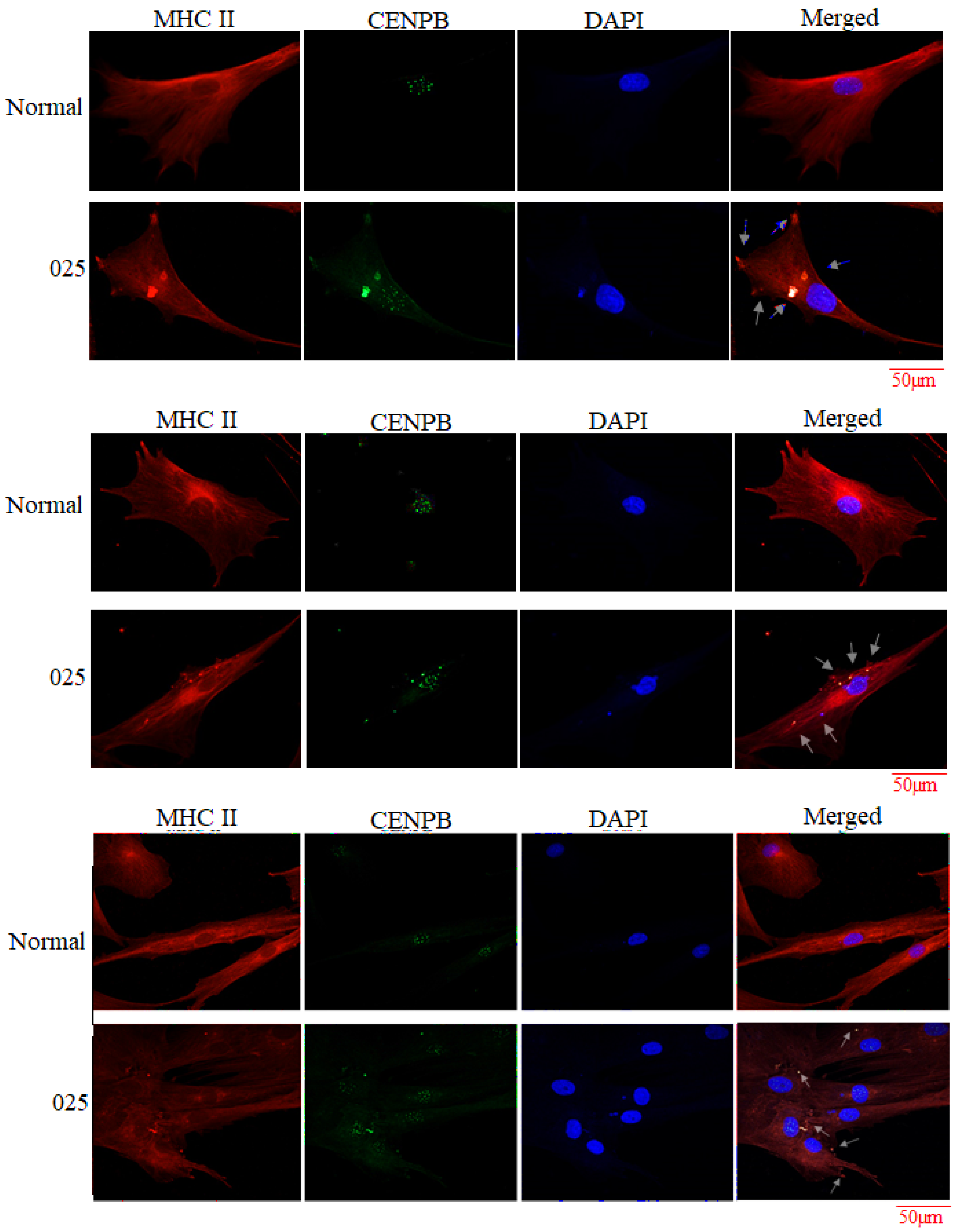

Supplementary Fig. 7. Colocalization of cytoplasmic CENPB and MHC Class II molecules in skin fibroblasts from a lcSSc patient with ACAs and a healthy individual. We performed IF to visualize colocalization of CENPB (green) and the expression of the MHC class II molecule DRB5 (beta 5 chain: red) in SSc skin fibroblasts. Nuclei were counterstained with DAPI (blue). Gray arrows indicate colocalization of cytoplasmic/membrane CENPB and MHCII in an lcSSc patient (025), who has ACAs antibodies. The scale bar is shown at the bottom right. MHC = major histocompatibility complex. Shown is a representation of at least 10 micrographs.

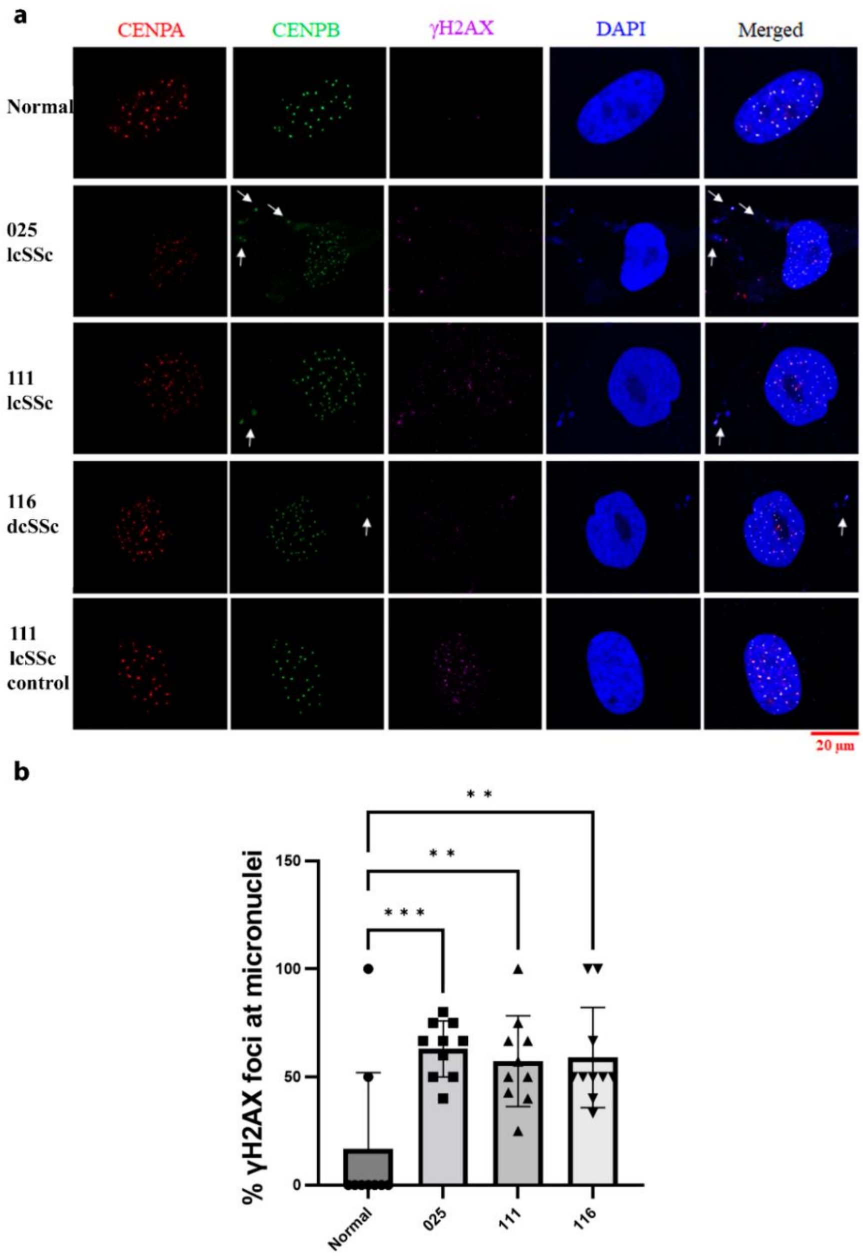

Supplementary Fig. 8. DNA damage to centromeres in SSc fibroblasts. a) We measured the expression and localization of γ-H2AX (purple), CENPA (red), and CENPB (green) in skin fibroblasts from lcSSc (025 and 111) and dcSSc (116) patients. DNA was counterstained with DAPI (blue). As a positive control for induced-DNA damage we performed serum starvation for 1 day in cultured skin fibroblasts from patient 111. Serum starvation produced an increased expression of γ-H2AX indicating DNA DSBs (111 lcSSc control). Arrows indicate colocalization of CENPB with γ-H2AX, micronuclei, and/or cytoplasmic DNA. b) The bar graph shows the percent of micronuclei stained with γ-H2AX in SSc patients compared to healthy skin fibroblasts (n = 10 micrographs for SSc fibroblasts, n = 9 micrographs for normal fibroblasts because of micronuclei scarcity). Data were analyzed using one-way ANOVA and Dunnett's multiple comparisons test. Normal vs. 025 $p = 0.0005$, Normal vs. 111 $p = 0.0022$, and Normal vs. 116 $p = 0.0015$. Data are presented as mean values +/- SD. Source Data are provided as a Source Data file.

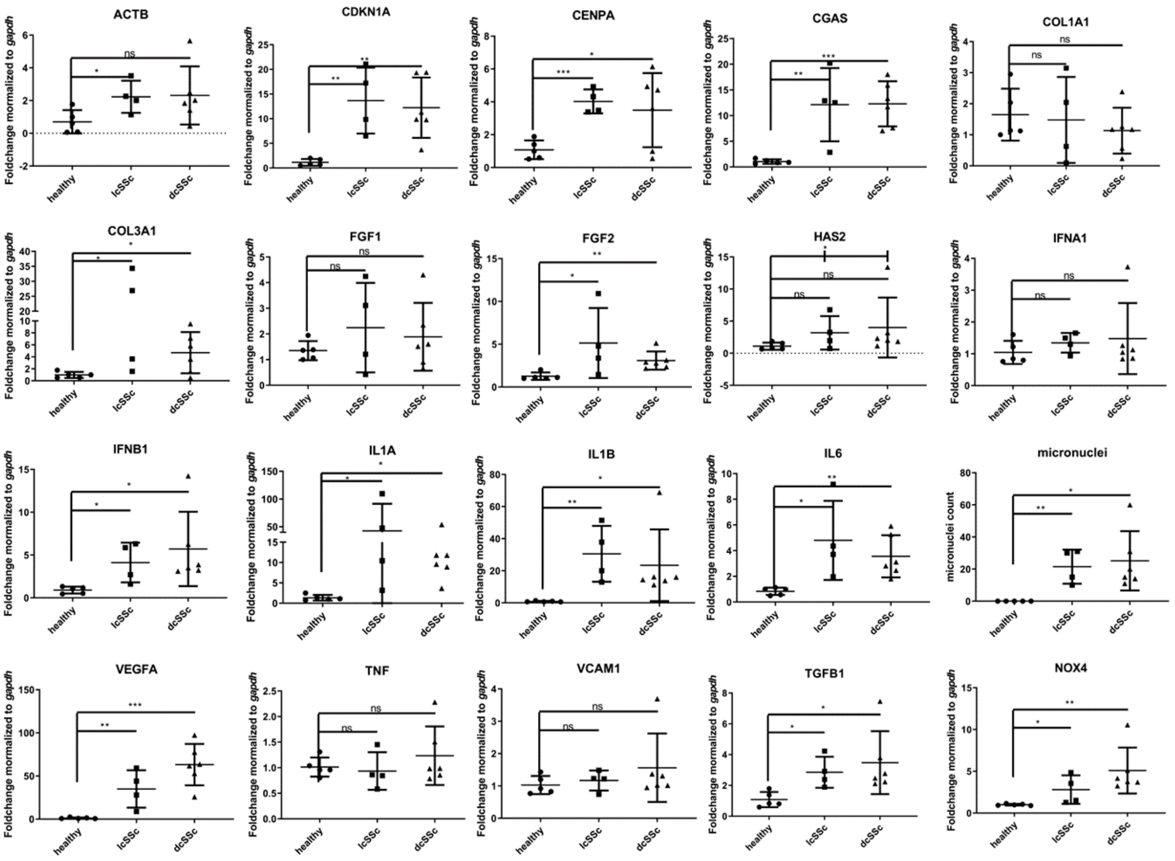

Supplementary Fig. 9. Expression levels of CENPA, profibrotic, proinflammatory, ROS, vasculopathy and cGAS-STING genes, and micronuclei counts in fibroblasts from SSc patients. The levels of RNA expression of *CENPA* and profibrotic and/or proinflammatory genes (*TGFB1, IL6; FGF1; FGF2; IL1A; IL1B; COL1A1; COL3A1, and TNF*), ROS genes (*CDKN1A, NOX4*), cGAS-STING (*cGAS, IFNA1, IFNB1, IL6*), and vasculopathy genes (*HAS2, VEGFA1, VCAM1*) were measured by qRT-PCR in 50 ng of DNase-treated RNA extracts and normalized to the levels of expression of the house keeping gene *GAPDH*, the levels of which did not vary among samples. The levels of RNA were compared between fibroblasts isolated from healthy dermal fibroblast samples and from skin lesions from patients with lcSSc and dcSSc. Differences in the number of micronuclei found in 500 nuclei counts are shown as well. Stars indicate statistically significant differences between the healthy individuals and lcSSc or dcSSc groups in a one-sided t-test analysis (* p = <0.5, ** p = <0.1, *** p = <0.001, **** p = < 0.0001, ns = not significance). Data are presented as mean values +/- SD. The exact p values can be found in the Source Data file. Source Data are provided as a Source Data file.

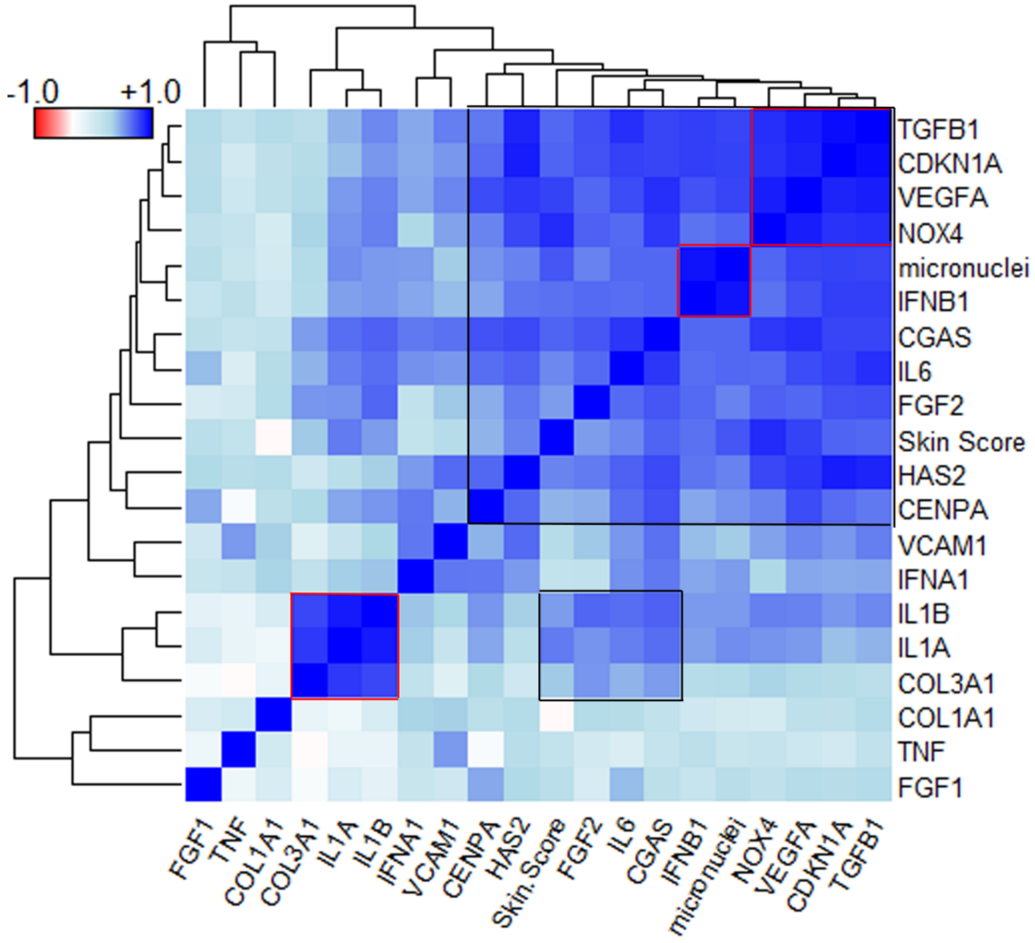

Supplementary Fig. 10. Correlation analysis between micronuclei and Modified Rodnan Skin Score and RNA expression. The Heatmap represents the Spearman Correlation R values between the variables. Black squares represent moderate correlation and red squares strong correlation values. Unsupervised hierarchical clustering analysis was conducted to ascertain if distinct genetic and genome instability signatures correlate with fibrotic, proinflammatory, vasculopathy, ROS, or cGAS-STING pathway genes. The correlation coefficients are color-coded from deep red (-1.0) to deep blue (+1.0). The presence of micronuclei and the expression of cGAS, the angiogenic gene VEGFA, and the pro-fibrotic gene TGFB1 all clustered strongly together (black and red boxes, upper right corner of figure). Expression of ROS genes (CDKN1A, and NOX4) also clustered strongly with expression of the fibrotic gene TGFB1 and the vasculopathy gene VEGFA (red box upper right corner). Expression of profibrotic genes COL3A1, IL1A, and IL1B clustered strongly together (red box, bottom left corner of figure). Source Data are provided as a Source Data file.

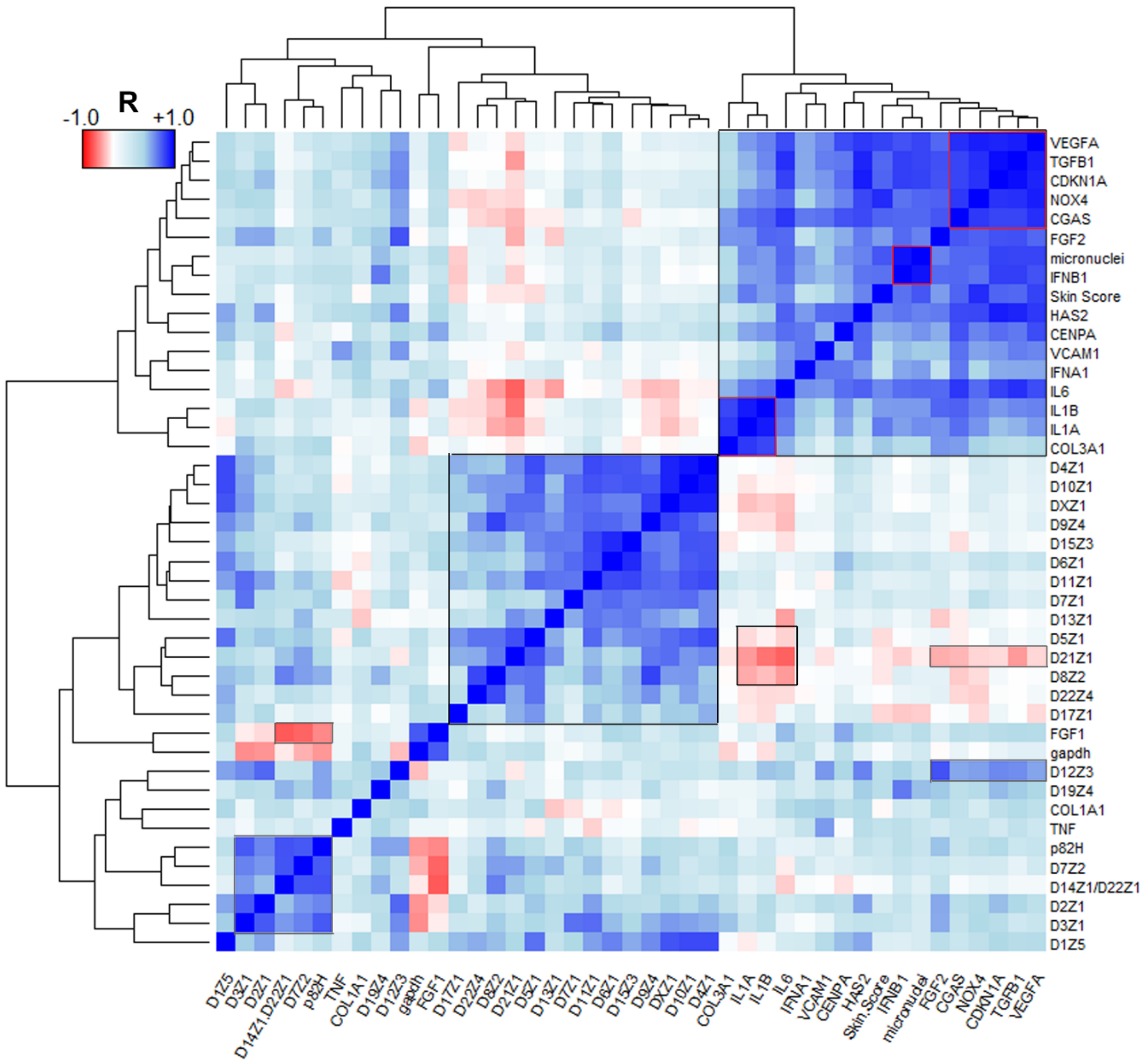

Supplementary Fig. 11. Correlation analysis between centromere size, chromosome instability (micronuclei), Modified Rodnan Skin Score, and RNA expression. The heatmap represents the Spearman Correlation R values between the variables. The black squares represent moderate correlation and red squares strong correlation values. Unsupervised hierarchical clustering analysis was conducted to ascertain if different genetic, chromosome instability signatures, and Modified Rodnan Skin scores correlate with fibrotic, proinflammatory, vasculopathy, ROS, or cGAS-STING pathway genes. The correlation coefficients are color-coded from deep red (-1.0) to deep blue (+1.0). The data are derived from the same patients as shown in Fig. 5. The presence of micronuclei and the expression of cGAS, the angiogenic gene VEGF, and the pro-fibrotic gene TGFB1 all clustered strongly together (black and red boxes, upper right corner of figure). Substantial loss of centromeric DNA was also seen in SSc, and loss of centromeric DNA in one chromosome often correlated with loss in another chromosome (large box in the center of figure) and some centromeres were largely missing (smaller box in the lower left-hand corner of figure). Intriguingly, loss of centromeric DNA in specific centromeres (from chromosomes 5, 8, and especially 21) was found to strongly correlate with the expression of key SSc genes (two red boxes in the middle of the right side of figure). Source Data are provided as a Source Data file.

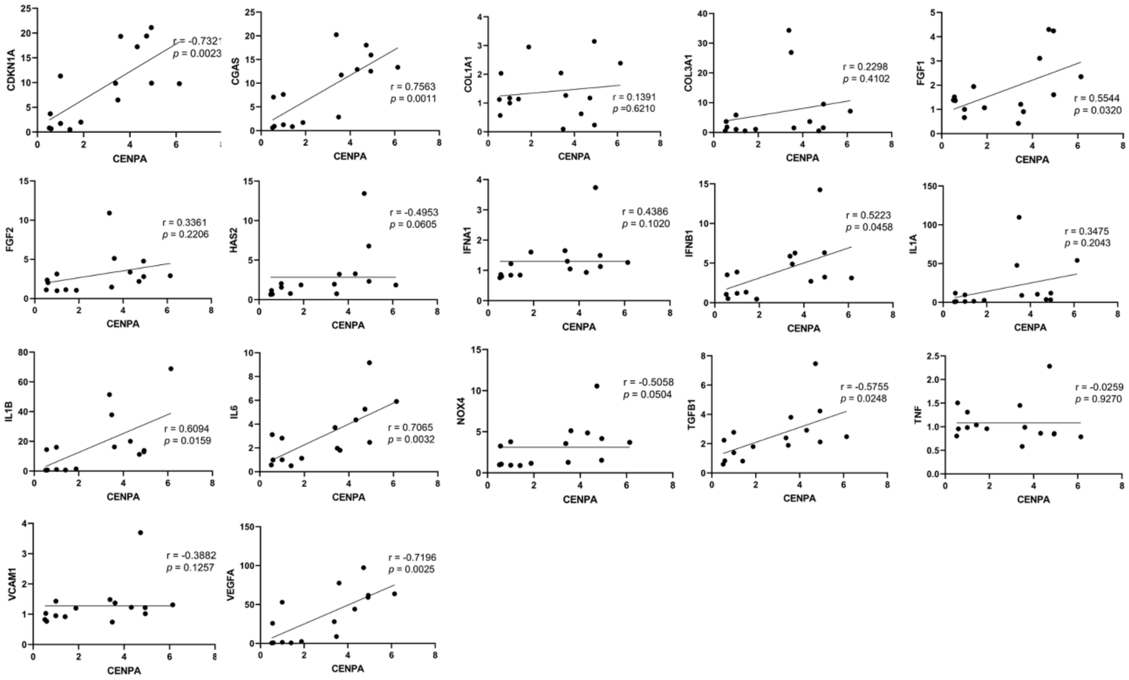

Supplementary Fig. 12. Correlations between the expression levels of CENPA in SSc fibroblasts and fibrotic, proinflammatory, ROS, vasculopathy and cGAS-STING genes in SSc fibroblasts. The levels of RNA expression of CENPA and profibrotic and/or proinflammatory genes (*TGFB1*, *IL6; FGF1; FGF2; IL1A; IL1B; COL1A1; COL3A1*, and *TNF*), ROS genes (*CDKN1A*, *NOX4*), cGAS-STING (*cGAS*, *IFNA1*, *IFNB1*, *IL6*), vasculopathy genes (*HAS2*, *VEGFA1*, *VCAM1*) were measured by qRT-PCR in 50 ng of DNase-treated RNA extracts and normalized to the levels of expression of the housekeeping gene *GAPDH*, the levels of which did not vary among samples. Shown are the *p* and r values calculated by one-sided Pearson statistical analysis. Source Data are provided as a Source Data file.

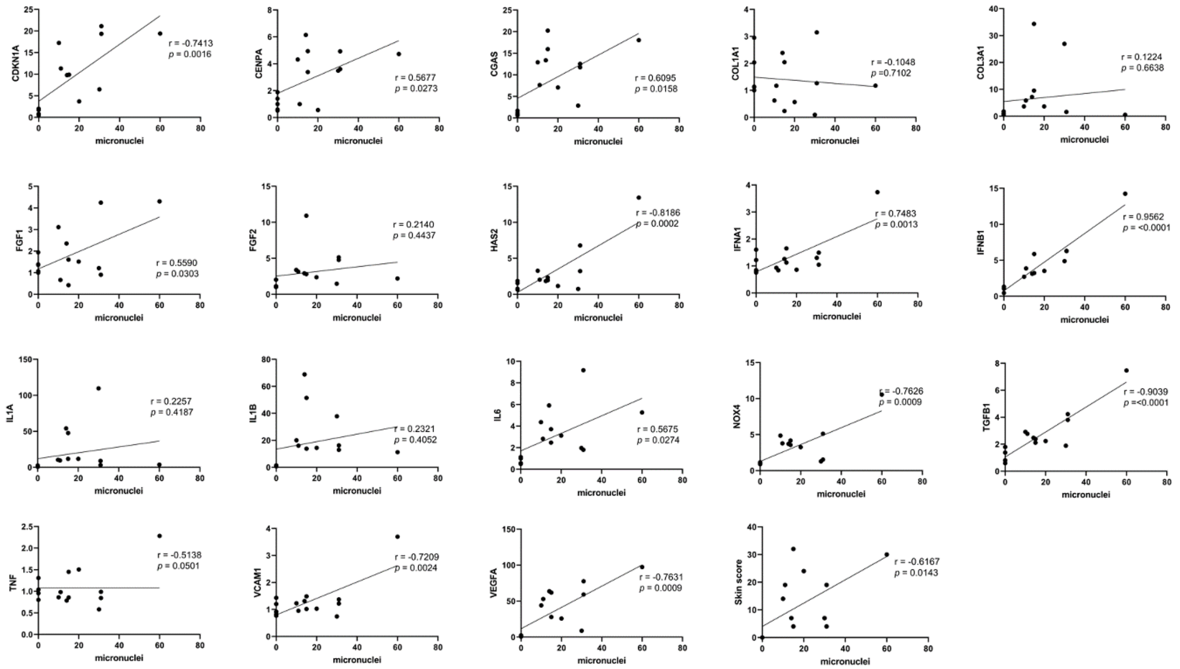

Supplementary Fig. 13. Correlations between the number of micronuclei and levels of expression of CENPA, fibrotic, proinflammatory, ROS, vasculopathy and cGAS-STING genes, and the Modified Rodnan Skin Score in SSc patients. The levels of RNA expression of *CENPA* and profibrotic and/or proinflammatory genes (*TGFB1, IL6; FGF1; FGF2; IL1A; IL1B; COL1A1; COL3A1*, and *TNF*), ROS genes (*CDKN1A*, *NOX4*), cGAS-STING genes (*cGAS*, *IFNA1*, *IFNB1*, *IL6*), vasculopathy genes (*HAS2*, *VEGFA1*, *VCAM1*) were measured by qRT-PCR in 50 ng of DNase-treated RNA extracts and normalized to the levels of expression of the housekeeping gene *GAPDH*, the levels of which did not vary among samples. Shown are the *p* and r values calculated by one-sided Pearson statistical analysis. Source Data are provided as a Source Data file.

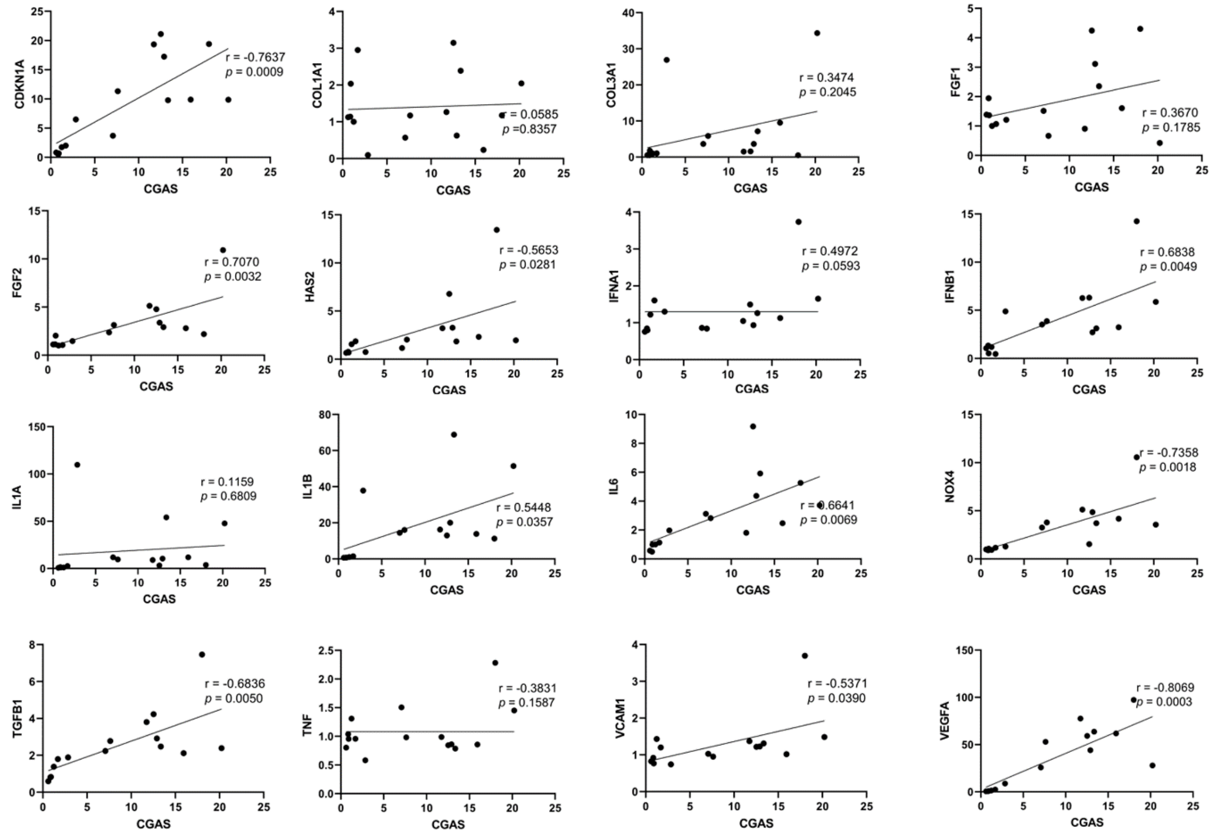

Supplementary Fig. 14. Correlations between the expression levels of cGAS and levels of expression of fibrotic, proinflammatory, ROS, and vasculopathy genes in SSc patients. The levels of RNA expression of *cGAS* and profibrotic and/or proinflammatory genes (*TGFB1, IL6; FGF1; FGF2; IL1A; IL1B; COL1A1; COL3A1*, and *TNF*), ROS genes (*CDKN1A, NOX4*), cGAS-STING genes (*IFNA1*, *IFNB1*, *IL6*), vasculopathy genes (*HAS2*, *VEGFA1*, *VCAM1*) were measured by qRT-PCR in 50 ng of DNase-treated RNA extracts and normalized to the levels of expression of the housekeeping gene *GAPDH*, the levels of which did not vary among samples. Shown are the *p* and r values calculated by one-sided Pearson statistical analysis. Source Data are provided as a Source Data file.

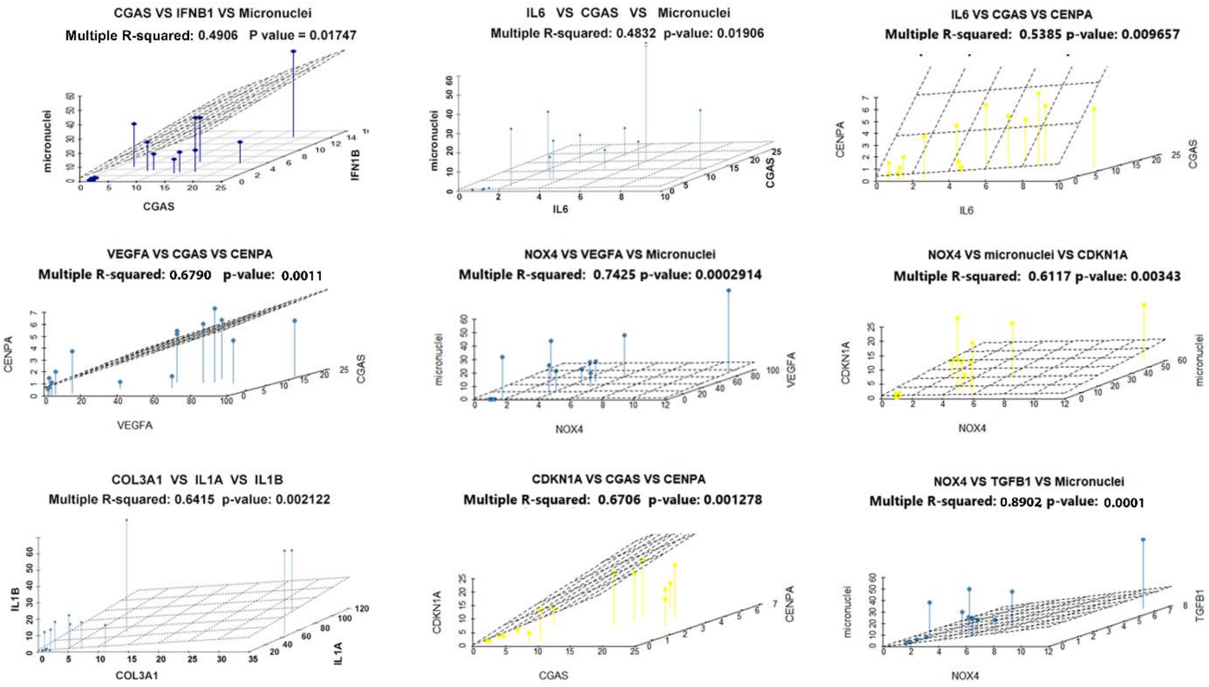

Supplementary Fig. 15. 3D Scatter plot analysis depicting the most significant associations between variables clustering in the heatmap shown in Fig. 5. Data were analyzed using multiple linear regression of Least squares assuming gaussian distribution of residuals. The multiple R-squared and *p* values for the three variables are shown in each graph. For example, the plot *NOX4* vs *TGFB1* vs Micronuclei in the right bottom graph shows an R-squared value of 0.8902, meaning a correlation coefficient r of 0.9435, indicating a strong positive correlation between the three variables. Therefore, an increase in the expression of *NOX4* and number of micronuclei positively correlates with the expression *TGFB1* in SSc patients (p <0.0001). Source Data are provided as a Source Data file.

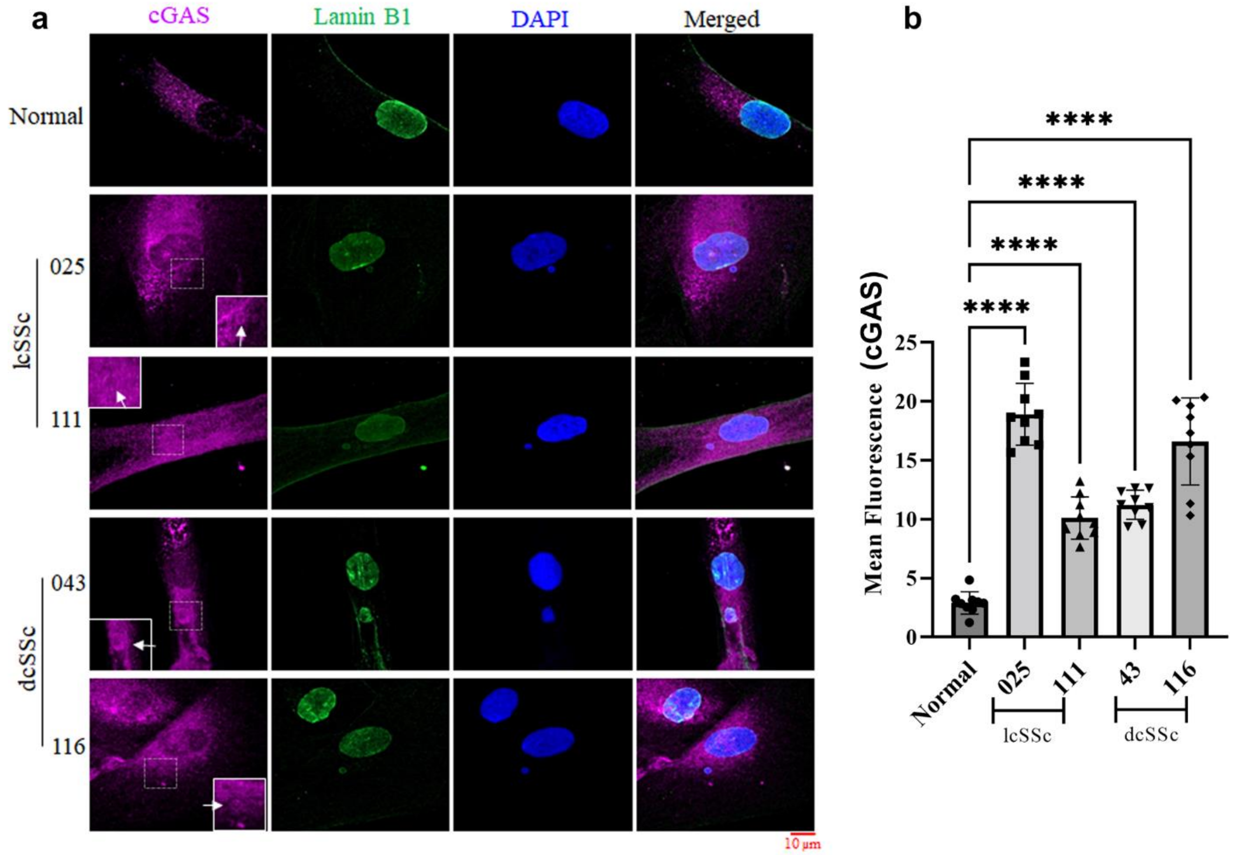

Supplementary Fig. 16. Expression of cGAS and co-localization to micronuclei in SSc skin fibroblasts. a) IF was performed to detect the level of expression of cGAS (purple), and the co-localization of cGAS to micronuclei in skin fibroblasts from lcSSc (025 and 111) and dcSSc (043 and 116) patients. Lamin B (green) detected the integrity of the nuclear membrane. Nuclei/micronuclei were counterstained with DAPI (blue). The scale bar is shown at the bottom right. Left panels show cGAS expression around micronuclei (squares). b) The bar graph shows the mean fluorescence level of expression of cytoplasmic cGAS in SSc patients compared to healthy skin fibroblasts (n= 9 micrographs). Data were analyzed using one-way ANOVA and Dunnett's multiple comparisons test. **** = $p < 0.0001$. Data are presented as mean values +/- SD. Source Data are provided as a Source Data file.

Supplementary Table 1. Demographics, laboratory evaluation, and studies of skin fibroblasts of patients with limited cutaneous (lc) or diffuse cutaneous (dc) SSc.

Study #	Disease	Age range	Disease onset	Skin score	ACAs	ATAs	RNA pol III antibodies	Past immunosuppressive therapy	Immunosuppressive therapy at time of sample	Cytoplasmic CENP-A/B (n=500)	Karyotype (n=20) # of abnormal spreads	micronuclei (n=500)*/(CENP B only micronuclei) **	Nuclear defects ***
17	lcSSc	65-70	1977	4	+	-	-	no	no	yes, 42	normal	10	yes
19	lcSSc	55-60	2003	2	+	ND	ND	MTX	no	yes, 167	normal	6	yes
25	lcSSc	65-70	1983	4	+	-	ND	no	no	yes, 392	normal	31 (5) **	yes
18	lcSSc	50-55	2012	7	-	-	-	MTX	MTX	no	normal	28 (2) **	yes
102	lcSSc	50-55	2010	4	+	ND	ND	MTX, HCQ	MTX, HCQ	yes, 60	normalmethotrexate Plaquenil	15	yes
108	lcSSc	45-50	2012	0	-	-	-	no	no	no	normal	10	yes
109	lcSSc	55-60	2010	6	+	-	ND	no	no	yes, 38	normal	17	yes
111	lcSSc	40-45	2007	7	+	-	-	AZA	no	yes, 20	normal	30 (2) **	yes
113	lcSSc	55-60	2014	14	-	-	-	MTX	CP, Tofacitinib	no	normal	10	yes
43	dcSSc	60-65	2012	24	-	-	-	RTX	MTX	no	aneuploidy, 4	20 (3) **	yes
60	dcSSc	40-45	2012	17	-	-	-	no	no	no	aneuploidy, 9	6	yes
65	dcSSc	50-55	2012	27	ND	+	ND	MTX, MMF	MMF	no	aneuploidy, 10	11	yes
74	dcSSc	70-75	2012	25	-	-	-	CP, RTX, MMF	MMF	no	aneuploidy, 3	9 (2) **	yes
103	dcSSc	55-60	2016	34	-	-	-	CP, HCQ	CP, HCQ	no	aneuploidy,12	2	yes
110	dcSSc	20-25	2014	19	ND	ND	ND	HCQ	MTX, Tofacitinib	no	aneuploidy, 2	11	yes
112	dcSSc	20-25	2010	32	-	+	-	MTX, HCQ, CP	no	no	normal	15 (4) **	yes
114	dcSSc	55-60	2017	30	-	-	+	Prednisone, MTX	RTX	no	aneuploidy, 10	60 (7) **	yes
115	dcSSc	55-60	2012	7	-	-	-	CP, Toclizumab	CP	no	aneuploidy, 4	14 (3) **	yes
116	dcSSc	55-60	2016	14	-	-	-	no	CP	no	aneuploidy, 10	22	yes
117	dcSSc	70-75	2016	19	-	-	-	Prednisone, CP	RTX	no	aneuploidy, 12	31 (4) **	yes

*Chromosome instability (CIN) was assessed by the presence of lagging chromosomes or micronuclei. ** Micronuclei stained with CENP-B but not CENP-A antibodies. *** Nuclear defects include convoluted and lobed nuclei. MTX=methotrexate, HCQ: Hydroxychloroquine, AZA: Azathioprine, RTX=rituximab, CP=cyclophosphamide, MMF=mycophenolate mofetil, F=female, M=Male, C=Caucasian, ND=not determined. ACAs= anti-centromere antibodies. ATAs=anti-topoisomerase antibodies. MTX=methotrexate. RTX=rituximab. MMF=mycophenolate mofetil. CP= cyclophosphamide. The skin sample of patient 108 with mRSS of “0” was taken from a lcSSc patient without skin thickness involvement. The patients’ fibroblasts were only used for cytogenetic analysis. The patients’ sex included 3 female/ 6 male lcSSc patients and 9 female/ 2 male dcSSc patients.

Supplementary Table 2. Demographics of SSc patients, laboratory analysis, immuno-suppressive therapy, and centromere/kinetochore and chromosome segregation assessment in monocyte-derived macrophages

Study #	Skin score	ACAs	ATAs	RNA pol III	Past immunosuppressive therapy	Immunosuppressive therapy at time of sample	Cytoplasm CENP-A/B (n=500)	micronuclei (n=500) ***	Nuclear defects ****	Extrachromosomal DNA
17	4	+	-	-	no	no	NA	NA	NA	NA
19	2	+	ND	ND	MTX	no	NA	NA	NA	NA
25	4	+	-	ND	no	no	NA	NA	NA	NA
18	7	-	-	-	MTX	MTX	NA	NA	NA	NA
102	4	+	ND	ND	MTX, HCQ	MTX, HCQ	methotrexate Plaquenil no	no	no	no
108	0	-	-	-	no	no				
109	6	+	-	ND	no	no	no	no	no	no
111	7	+	-	-	AZA	no	no	no	no	no
113	14	-	-	-	MTX	CP, Tofacitinib	no	no	no	no
43	24	-	-	-	RTX 11/2013	MTX	no	no	no	no
60	17	-	-	-	no	no	no	no	no	no
65	27	ND	+	ND	MTX, MMF	MMF	NA	NA	NA	NA
74	25	-	-	-	CP, RTX, MMF	MMF	NA	NA	NA	NA
103	34	-	ND	+	CP, HCQ	CP, HCQ	no	no	no	no
110	19	ND	ND	ND	HCQ	MTX, Tofacitinib	no	no	no	no
112	32	-	+	-	MTX, HCQ, CP	no	no	no	no	no
114	30	ND	ND	ND	Prednisone, MTX	RTX	no	no	no	no
115	7	-	-	-	CP, Toclizumab	CP	no	no	no	no
116	14	-	-	-	no	CP	no	no	no	no
117	19	ND	ND	-	Prednisone, CP	RTX	no	no	no	no

*** Chromosome instability (CIN) was assessed by the presence of lagging chromosomes or micronuclei. **** Nuclear defects include convoluted and lobed nuclei. MTX=methotrexate, HCQ: Hydroxychloroquine, AZA: Azathioprine, RTX=rituximab, CP=cyclophosphamide, MMF=mycophenolate mofetil, F=female, M=Male, C=Caucasian, ND=not determined. NA: not available. ACAs= anti-centromere antibodies. ATAs=anti-topoisomerase antibodies. RNA pol III= anti-RNA pol III antibodies.

Supplementary Table 3. Demographics of SSc patients, laboratory analysis, immuno-suppressive therapy, and centromere/kinetochore and chromosome segregation assessment in peripheral blood lymphocytes

Study #	Skin score	ACAs	ATAs	RNA pol III	Past immunosuppressive therapy	Immunosuppressive therapy at time of sample	Cytoplasm CENP-A/B (n=500)	Karyotype* (n=20)	micronuclei (n=500) ***	Nuclear defects ****
17	4	+	-	-	no	no	NA	NA	NA	NA
19	2	+	ND	ND	MTX	no	NA	NA	NA	NA
25	4	+	-	ND	no	no	NA	NA	NA	NA
18	7	-	-	-	MTX	MTX	NA	NA	NA	NA
102	4	+	ND	ND	MTX, HCQ	MTX, HCQ	no	normal	no	no
108	0	-	-	-	no	no				
109	6	+	-	ND	no	no	no	normal	no	no
111	7	+	-	-	AZA	no	no	normal	no	no
113	14	-	-	-	MTX	CP, Tofacitinib	no	normal	no	no
43	24	-	-	-	RTX 11/2013	MTX	no	normal	no	no
60	17	-	-	-	no	no	no	normal	no	no
65	27	ND	+	ND	MTX, MMF	MMF	NA	NA	NA	NA
74	25	-	-	-	CP, RTX, MMF	MMF	NA	NA	NA	NA
103	34	-	ND	+	CP, HCQ	CP, HCQ	no	normal	no	no
110	19	ND	ND	ND	HCQ	MTX, Tofacitinib	no	normal	no	no
112	32	-	+	-	MTX, HCQ, CP	no	no	normal	no	no
114	30	ND	ND	ND	Prednisone, MTX	RTX	no	normal	no	no
115	7	-	-	-	CP, Toclizumab	CP	no	normal	no	no
116	14	-	-	-	no	CP	no	normal	no	no
117	19	ND	ND	-	Prednisone, CP	RTX	no	normal	no	no

*NA: Karyotype was not done for slow-growing cells that could not be arrested in metaphase with colchicine. *** Chromosome instability (CIN) was assessed by the presence of lagging chromosomes or micronuclei. **** Nuclear defects include convoluted and lobed nuclei MTX=methotrexate, HCQ: Hydroxychloroquine, AZA: Azathioprine, RTX=rituximab, CP=cyclophosphamide, MMF=mycophenolate mofetil, F=female, M=Male, C=Caucasian. ND=not determined.

Supplementary Table 4. List of Primers

GENE	FORWARD (5' TO 3')	REVERSE (5' TO 3')
**CDKN1A**	GAGACTAAGGCAGAAGATGTAGAG	GCAGACCAGCATGACAGAT
**CENPA**	CAAAGGATGTGCAACTGGC	CATTGGATCTAGTCATGGCTCTG
**CGAS**	GGGAGCCCTGCTGTAACACTTCTTAT	CCTTTGCATGCTTGGGTACAAGGT
**COL1A1**	TTCACCTACAGCACGCTTGT	TTGGGATGGAGGGAGTTTAC
**COL3A1**	GGTCACTTTCACTGGTTGACGA	TTGAATATCAAACACGCAAGGC
**FGF1**	ACAAGGGACAGGAGCGAC	TCCAGCCTTTCCAGGAACA
**FGF2**	ATGGCAGCCGGGAGCATCACCCACG	TCAGCTCTTCGCAGACATTGGAAG
**HAS2**	GCCTCATCTGTGGAGATGGT	ATGCACTGAACACACCCAAA
**IFNA**	GACTCCATCTTGGCTGTGA	TGATTTCTGCTCTGACAACCT
**IFNB**	TTGACATCCCTGAGGAGATTAAGC	TTAGCCAGGAGGTTCTCAACAATAG
**IL1A**	CGAGCCAATGATCAGTACCTC	GGTAGTGTCCATCACTCTGG
**IL1B**	GGCAGACTCAAATTCCAGCT	ACAACGAGGTATAGGACAGG
**IL6**	CAGGAACTGGATCAGGACTT	GACGAAAGTGTGTACAATGA
**NOX4**	ATCTGGCTCTCCATGAATGTCCTG	ACACAATCCTAGCCCCAACATCTG
**TNF**	CAACCTCTTCTGGCTCAA	CGAAGTGGTGGTCTTGTT
**TGFB**	GCCCTGGACACCAACTATTG	GTCCAGGCTCCAAATGTAGG
**VCAM1**	GATACAACCGTCTTGGTCAGCCC	CAGTTGAAGGATGCGGGAGTATATG
**VEGFA1**	GTTGACCTTCCTCCATCC	TTCTCTGCCTCCACAATG

Supplementary Figure 6. Original Western blots

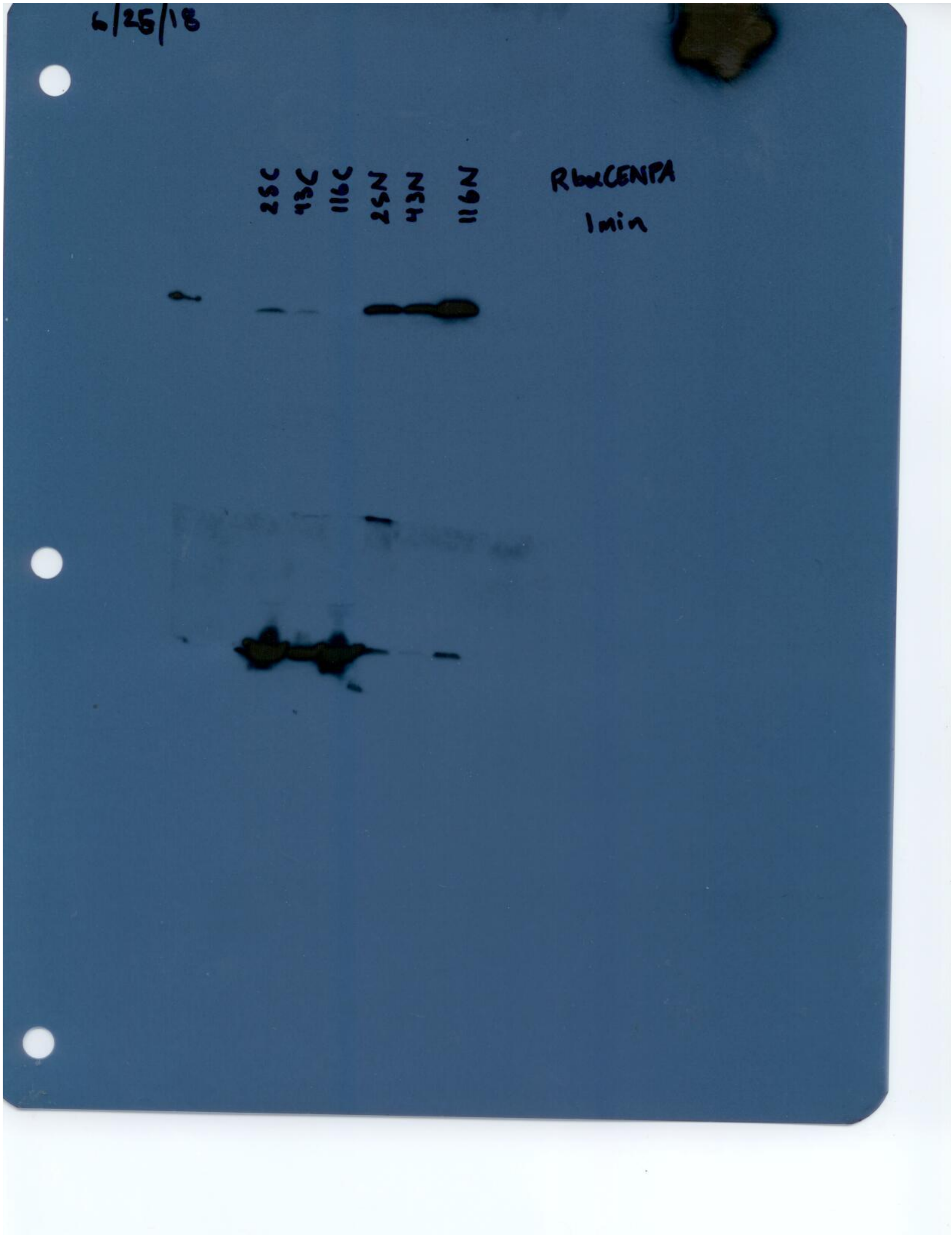

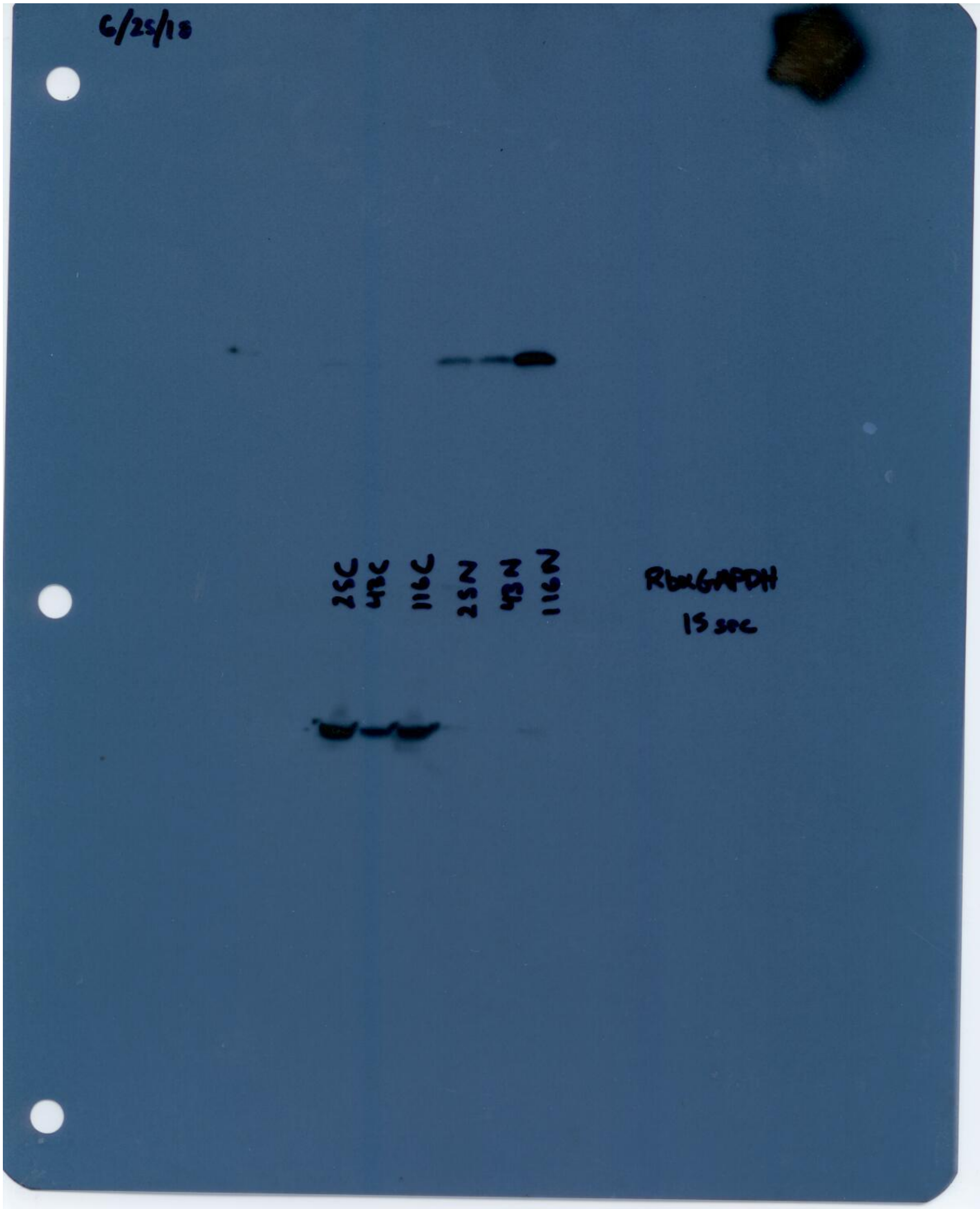
6/25/18
25C
116C
25N
116N
RbαGAPDH
15 sec

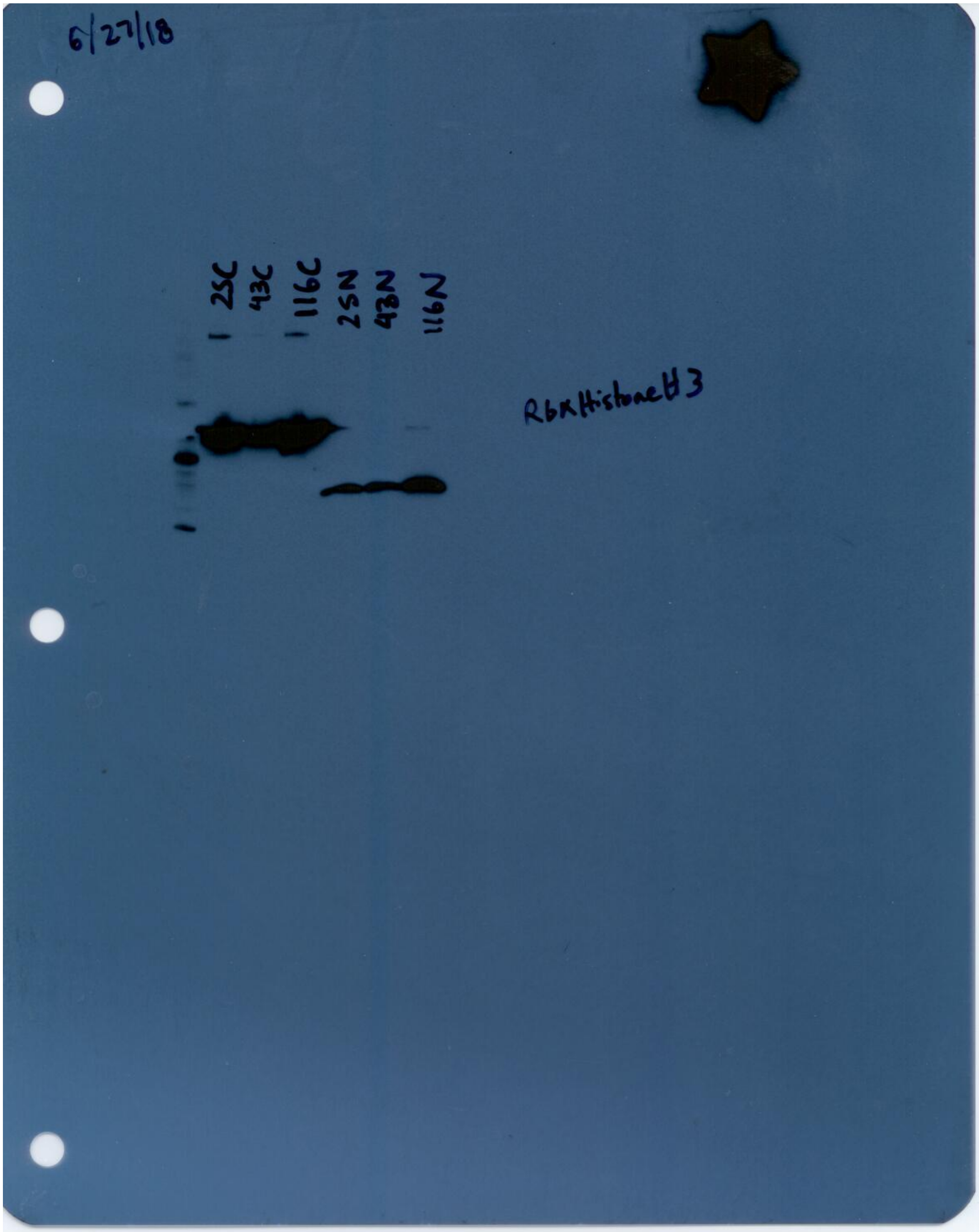
6/27/18
25C
43C
116C
25N
43N
116N
RbxHistoneH3
