# Supplementary material for: Centromere defects, chromosome instability, and cGAS-STING activation in systemic sclerosis
Source: Nat Commun. 2022 Nov 18;13:7074. doi: 10.1038/s41467-022-34775-8 (PMC9674829; doi:10.1038/s41467-022-34775-8)
Supplement: Supplementary file 2 — Reporting Summary [file 41467_2022_34775_MOESM2_ESM.pdf]

nature portfolio

Corresponding author(s): Rafael Contreras-Galindo
David M Markovitz

Last updated by author(s): 2022/08/31

Reporting Summary

Nature Portfolio wishes to improve the reproducibility of the work that we publish. This form provides structure for consistency and transparency in reporting. For further information on Nature Portfolio policies, see our Editorial Policies and the Editorial Policy Checklist.

Statistics

For all statistical analyses, confirm that the following items are present in the figure legend, table legend, main text, or Methods section.

n/a	Confirmed	
☐	☒	The exact sample size (*n*) for each experimental group/condition, given as a discrete number and unit of measurement
☒	☐	A statement on whether measurements were taken from distinct samples or whether the same sample was measured repeatedly
☐	☒	The statistical test(s) used AND whether they are one- or two-sided<br>*Only common tests should be described solely by name; describe more complex techniques in the Methods section.*
☒	☐	A description of all covariates tested
☒	☐	A description of any assumptions or corrections, such as tests of normality and adjustment for multiple comparisons
☐	☒	A full description of the statistical parameters including central tendency (e.g. means) or other basic estimates (e.g. regression coefficient) AND variation (e.g. standard deviation) or associated estimates of uncertainty (e.g. confidence intervals)
☐	☒	For null hypothesis testing, the test statistic (e.g. *F*, *t*, *r*) with confidence intervals, effect sizes, degrees of freedom and *P* value noted<br>*Give P values as exact values whenever suitable.*
☒	☐	For Bayesian analysis, information on the choice of priors and Markov chain Monte Carlo settings
☒	☐	For hierarchical and complex designs, identification of the appropriate level for tests and full reporting of outcomes
☐	☒	Estimates of effect sizes (e.g. Cohen's *d*, Pearson's *r*), indicating how they were calculated

Our web collection on statistics for biologists contains articles on many of the points above.

Software and code

Policy information about availability of computer code

Data collection	No software was used.
Data analysis	GraphPad Prism version 9.0.0 for Windows and R Studio Version 1.4.1103 gplots, RcolorBrewer, and plotrix packages

For manuscripts utilizing custom algorithms or software that are central to the research but not yet described in published literature, software must be made available to editors and reviewers. We strongly encourage code deposition in a community repository (e.g. GitHub). See the Nature Portfolio guidelines for submitting code & software for further information.

Data

Policy information about availability of data

All manuscripts must include a data availability statement. This statement should provide the following information, where applicable:

- Accession codes, unique identifiers, or web links for publicly available datasets
- A description of any restrictions on data availability
- For clinical datasets or third party data, please ensure that the statement adheres to our policy

Source data are provided with this paper

Field-specific reporting

Life sciences study design

All studies must disclose on these points even when the disclosure is negative.

Sample size	Sample size was determined from standard practice in the field and was sufficient to perform statistical analysis
Data exclusions	The skin sample of patient 108 with mRSS of "0" was taken from a lcSSc patient without skin thickness involvement. The patients' fibroblasts were only used for cytogenetic analysis. No data were excluded.
Replication	Each experiment was performed at least three times unless otherwise indicated. All replications were successful.
Randomization	Not applicable -we did not use distinct groupings of patients across which patients could be randomized
Blinding	Samples were collected de-identified to the PI and the researchers.<br>Investigators did not have clinical information and in that sense, the experiments were performed blinded.<br>Fluorescence signal measurements were performed using the open-source software ImageJ (NIH). Thus, no personal or manual judgment was made that could bias the data.

Reporting for specific materials, systems and methods

We require information from authors about some types of materials, experimental systems and methods used in many studies. Here, indicate whether each material, system or method listed is relevant to your study. If you are not sure if a list item applies to your research, read the appropriate section before selecting a response.

Materials & experimental systems

n/a	Involved in the study	
☐	☒	Antibodies
☒	☐	Eukaryotic cell lines
☒	☐	Palaeontology and archaeology
☒	☐	Animals and other organisms
☐	☒	Human research participants
☒	☐	Clinical data
☒	☐	Dual use research of concern

Methods

n/a	Involved in the study	
☒	☐	ChIP-seq
☒	☐	Flow cytometry
☒	☐	MRI-based neuroimaging

Antibodies

Antibodies used	Primary Antibodies: anti-CENPA (MBL international 1:500, Code # D115-3), anti-CENPB primary antibody (Santa Cruz Biotechnology, CENPB Antibody (C-10) 1:50: sc-376392, pSer139-γ-H2AX antibody (Cell Signaling 1:400, Cat # 9718), BANF1/BAF antibody (abcam,1:100, EPR7668), MHCII DRB1 antibody (Abclonal, 1:100, Cat # A7685), Lamin B1 antibody (Proteintech, 1:1000, Cat # 66095-1-lg), cGAS antibody (Abclonal, 1:100, Cat# A8335), p(Ser396)-IRF antibody (Cell Signaling, 1:100, Cat # 29047), p(Ser536)-p65 antibody (Abclonal, 1:100, Cat # AP0123), MHCII DRB5 antibody (Abclonal, 1:100, Cat # A12726), anti-beta actin antibody (Abcam, ab227387), anti-GAPDH antibody (Abcam, ab 9484), anti-Histone H3 (tri methyl K9) antibody (Abcam, ab8898), anti-CENPA antibody [3-19] (Abcam, ab13939)<br><br>Secondary Antibodies: Alexa Flour 594 rabbit anti-mouse IgG 1:1000, Thermo Fischer Scientific, Cat No A27027, Alexa Flour 488 rabbit anti-mouse IgG 1:1000, Thermo Fischer Scientific, Cat No A27023. Alexa fluor 647 Goat anti-rabbit IgG, ThermoFisher, 1:1000, Cat # A32733TR
Validation	Anti-CENP-A mAb 3-19 has been validated in several publications, including WB, IF, and IHC in human cell lines and tissues by the manufacturer. Further numerous articles have been used this antibody successfully in centromere research (Marshall OJ et al. J Cell Biol. 183, 1193-202 (2008))<br>Anti-CENP-B (C-10) has been validated in several publications, including WB, IP, IF, and ChIP in mouse, rat and human cells by the manufacturer. Further numerous articles have been used this antibody successfully in centromere research (Chunduri, N.K., et al. 2021. Systems approaches identify the consequences of monosomy in somatic human cells. Nat. Commun. 12: 5576.)<br>γ-H2AX antibody has been validated in several publications, including WB, IP, IF, and ChIP in several species by the manufacturer. Further numerous articles have been used this antibody successfully (Qin Wu, et. al. 2022. Nat. Chem. Biol. Aug;18(8):821-830)<br>BANF1/BAF antibody has been validated in several publications, including WB, IHC, IF, and FC in mouse, dog and human cells by the manufacturer. Further numerous articles have been used this antibody successfully (Essawy N et. al. Cells 8:N/A (2019).<br>MHCII DRB1 antibody has been validated in several publications, including WB, in human cells by the manufacturer. Validation data provided by the manufacturer: Western blot analysis of extracts of various cell lines, using HLA-DRB1 antibody (A7685) at 1:1000 dilution. Secondary antibody: HRP Goat Anti-Rabbit IgG (H+L) (AS014) at 1:10000 dilution._Lysates/proteins: 25ug per lane._Blocking buffer: 3% nonfat dry milk in TBST._Detection: ECL Basic Kit (RM00020)._Exposure time: 30s<br>Lamin B1 antibody has been validated in several publications, including WB, IP, IF, FC and IHC in human cells by the manufacturer. Further numerous articles have been used this antibody successfully (Yan Wanf, et. al. 2019. Nat. Commun. Feb 4;10(1):564)

cGAS antibody has been validated in IF by the manufacturer. Validation data provided by the manufacturer: Immunofluorescence analysis of PC-12cells using cGAS Rabbit pAb (A8335) at dilution of 1:50 (40x lens). Blue: DAPI for nuclear staining. Immunofluorescence analysis of U2OS cells using cGAS Rabbit pAb (A8335) at dilution of 1:50 (40x lens). Blue: DAPI for nuclear staining.
p(Ser396)-IRF antibody has been validated in several publications, including WB, IP, and IF, FC in mice and human cells by the manufacturer. Further numerous articles have been used this antibody successfully (Ayano Kabashima, et. al. 2022. Sci. Rep. Jun 30;12(1):10466)
p(Ser536)-p65 antibody has been validated in IF by the manufacturer. Validation data provided by the manufacturer: Western blot analysis of extracts of NIH/3T3 cells, using Phospho-NF-kB p65/RelA-S276 antibody (AP0123) at 1:1000 dilution.NIH/3T3 cells were treated by TNF-α (20 ng/ml) at 37C for 30 minutes. Secondary antibody: HRP Goat Anti-Rabbit IgG (H+L) (AS014) at 1:10000 dilution. Lysates/proteins: 25ug per lane. Blocking buffer: 3% nonfat dry milk in TBST. Detection: ECL Basic Kit (RM00020). Exposure time: 180s.
MHCII DRB5 antibody has been validated in IF by the manufacturer. Validation data provided by the manufacturer: Western blot analysis of extracts of various cell lines, using HLA-DRB5 antibody (A12726) at 1:3000 dilution. Secondary antibody: HRP Goat Anti-Rabbit IgG (H+L) (AS014) at 1:10000 dilution. Lysates/proteins: 25ug per lane. Blocking buffer: 3% nonfat dry milk in TBST. Detection: ECL Basic Kit (RM00020). Exposure time: 10s
anti-beta actin antibody has been validated in WB, IHC and IF by the manufacturer. Further numerous articles have been used this antibody successfully (Zhu X et al. J Bone Oncol 27:100347 (2021))
anti-GAPDH ab9484 antibody has been validated in WB, IHC and IF by the manufacturer. Further numerous articles have been used this antibody successfully (Gao S et al. Life Sci 266:118863 (2021))
anti-Histone H3 (tri methyl K9) ab8898 antibody has been validated in WB, IHC and IF by the manufacturer. Further numerous articles have been used this antibody successfully (Liang C et al. Cell Res 31:187-205 (2021))
anti-CENPA [3-19] ab13939 antibody has been validated in WB, IHC ChIP and IF by the manufacturer. Further numerous articles have been used this antibody successfully (KL Dale et al. J Cell Sci Sep 1;135(17): jcs260031 (2022))

Human research participants

Policy information about studies involving human research participants

Population characteristics

Table 1

Recruitment

The University of Michigan Medical Institutional Review Board (HUM00065044) approved the procedures described in this study and patients were recruited u in the University of Michigan Hospital, Scleroderma Clinic. Participant were given a verbal and written information about the study before their participation. Written informed consent was obtained from the participants before enrollment in the study. The study design and conduct complied with all relevant regulations regarding the use of human study participants and was conducted in accordance with the criteria set by the Declaration of Helsinki.
We included all the patients who agreed to participate.
Participants were Scleroderma patients with lcSSc and dcSSc diagnosis.
We did not find any self-selection bias in these criteria.
No participant compensation was given.

Ethics oversight

The University of Michigan Medical Institutional Review Board (HUM00065044)

Note that full information on the approval of the study protocol must also be provided in the manuscript.
